# Injury-induced ASCL1 expression orchestrates a transitory cell state required for repair of the neonatal cerebellum

**DOI:** 10.1126/sciadv.abj1598

**Published:** 2021-12-08

**Authors:** N. Sumru Bayin, Dogukan Mizrak, Daniel N. Stephen, Zhimin Lao, Peter A. Sims, Alexandra L. Joyner

**Affiliations:** 1 Developmental Biology Program, Sloan Kettering Institute, New York, NY, USA.; 2 Department of Systems Biology, Columbia University, New York, NY, USA.; 3 Department of Biochemistry and Biophysics, Columbia University, New York, NY, USA.; 4 Biochemistry, Cell and Molecular Biology Program, Weill Cornell Graduate School of Medical Sciences, New York, NY, USA.

## Abstract

To understand repair processes, it is critical to identify the molecular foundations underlying progenitor diversity and plasticity. Upon injury to the neonatal cerebellum, a normally gliogenic *nestin*-expressing progenitor (NEP) in the Bergmann glia layer (BgL) undergoes adaptive reprograming to restore granule cell production. However, the cellular states and genes regulating the NEP fate switch are unknown. Using single-cell RNA sequencing and fate mapping, we defined molecular subtypes of NEPs and their lineages under homeostasis and repair. NEPs contain two major subtypes: *Hopx*^+^ astrogliogenic and *Ascl1*^+^ neurogenic NEPs that are further subdivided based on their location, lineage, and differentiation status. Upon injury, an *Ascl1*^+^ transitory cellular state arises from *Hopx*^+^ BgL-NEPs. Furthermore, mutational analysis revealed that induction of *Ascl1* is required for adaptive reprogramming by orchestrating a glial-to-neural switch in vivo following injury. Thus, we provide molecular and cellular insights into context-dependent progenitor plasticity and repair mechanisms in the brain.

## Teaser

ASCL1 is induced in a glial progenitor by cerebellar injury and directs adaptive reprograming to replace granule neurons.

## INTRODUCTION

For complex tissues like the brain to be generated or to undergo repair, an effective cellular strategy involves having a multiplicity of progenitor subtypes and flexibility in fate choices. Development of single-cell approaches has been instrumental in dissecting progenitor diversity and identifying transitory cell states critical to fate decisions during development and to a lesser extent repair (*1*, *2*). The neonatal cerebellum has emerged as a valuable system to identify molecular mechanisms that drive plasticity after injury because of its high regenerative potential and diversity of progenitor populations. The cerebellum is a folded hindbrain structure that houses most of the neurons in the brain (*3*, *4*) and is important for motor and higher-order cognitive functions (*5*–*8*). It has prolonged development compared to the rest of the brain as production of most cerebellar cells occurs after birth in mammals. The postnatal progenitors in the folds/lobules of the cerebellum continue proliferating up to 6 months after birth in humans (*9*) and 2 weeks in mice (*10*). The late development of the cerebellum leads to increased susceptibility to injury around birth, and cerebellar hypoplasia is the second leading risk factor of autism spectrum disorders (*11*). The newborn rodent cerebellum can efficiently replenish at least two of its main cell types when they are ablated (*12*–*16*), and one repair process involves unexpected progenitor plasticity and an apparent glial-to-neural fate switch after injury. The molecular mechanisms that drive progenitor plasticity in vivo, however, are unknown in the neonatal cerebellum.

**Fig. 1.**
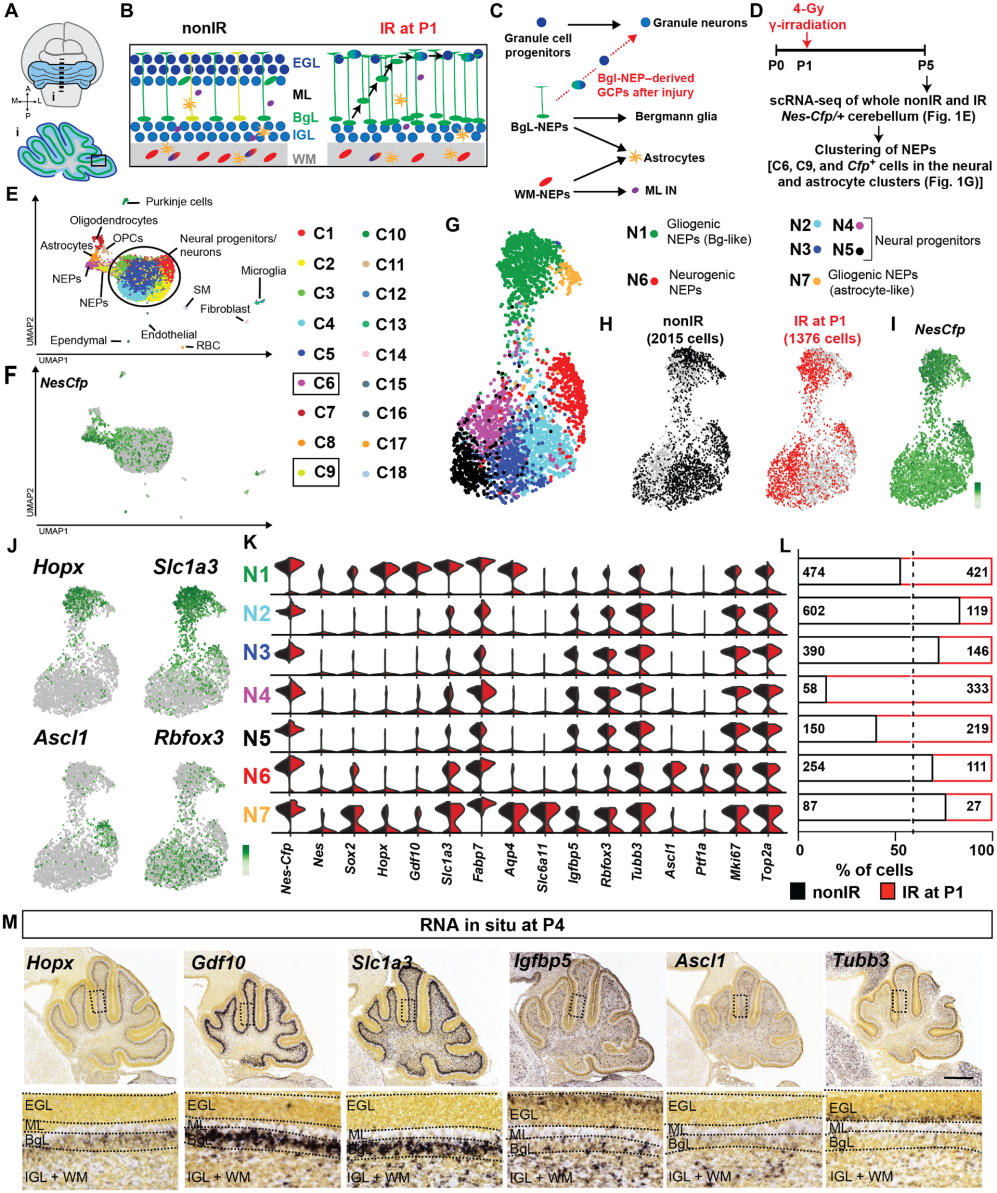
scRNA-seq of P5 cerebellar NEPs reveals molecular NEP subtypes. (**A** to **C**) Schematics showing the neonatal cerebellum, cell types in each layer, the previously proposed lineages of the postnatal cerebellar progenitors, and the changes that occur after injury (irradiation). (**D**) Experimental plan. (**E** and **F**) Uniform Manifold Approximation and Projection (UMAP) visualization of the clustering of 18,929 cells shown by cell types (E) and by *Nes-Cfp* expression (F). *Cfp*-enriched clusters [C6 and C9, log_2_ fold change > 1, false discovery rate (FDR) < 0.05] that contain NEPs are highlighted. (**G** and **H**) UMAP embedding of the clustering of 2015 nonIR and 1276 IR NEPs by cluster (G) and by sample (H). (**I** and **J**) Projection of the expression levels of *Nes-Cfp* (I) and lineage markers (J) on the UMAP [scale shows the log-transformed CPM (counts per million) where the darker colors show higher expression]. (**K**) Violin plots showing the log_10_CPM expression levels of the canonical lineage genes and some of the top significantly enriched genes in clusters shown in (G). (**L**) Number of nonIR and IR cells and their distribution within each cluster. Dashed line shows the percent distribution of nonIR and IR cells in all the cells. (**M**) Allen Brain Atlas P4 RNA in situ hybridization data showing the expression patterns of marker genes identified by scRNA-seq. Scale bar, 500 μm. SM, smooth muscle; RBC, red blood cell; ML, molecular layer; IGL, internal granule layer; OPC, oligodendrocyte progenitor cells.

After birth, several distinct cerebellar progenitor populations derived from the rhombic lip or the ventricular zone continue to proliferate and generate late-born cells in mouse. The rhombic lip–derived *Atoh1*-expressing granule cell progenitors (GCPs) proliferate in the external granular layer on the surface of the cerebellum and, upon their last cell division, produce excitatory neurons that migrate inward to form the inner granular layer (*17*–*19*). A poorly defined group of cerebellar ventricular zone–derived *nestin*-expressing progenitors (NEPs) in the lobules give rise to astrocytes, interneurons (INs), and Bergmann glia (Bg), a specialized polarized glial cell with fibers extending to the cerebellar surface (*12*, *20*–*24*). Fate mapping studies have indicated that the lineage propensity of NEPs depends on their location, such that NEPs in the layer that houses the Bg (BgL) or in the prospective white matter (WM) produce Bg or INs, respectively (*12*, *20*, *21*, *24*–*26*). It is less clear where the progenitors of astrocytes reside (Fig. 1, A to C). Furthermore, there are NEPs deep within the cerebellum in the WM surrounding the cerebellar nuclei (*24*). However, whether deep and lobule WM-NEPs are molecularly and functionally distinct is unknown. Thus, the full extent of the diversity of postnatal NEPs and the molecular signature, lineage propensity, and location of distinct NEP subtypes are unknown.

When the proliferating GCPs are depleted in the newborn cerebellum either via genetic approaches or by irradiation, the external granule layer (EGL) recovers within a week and the cerebellum grows to almost a normal size (*12*, *13*, *15*, *16*). A key aspect of the regenerative process involves BgL-NEPs undergoing adaptive reprograming to replenish the lost GCPs. The first response of the BgL-NEPs is an increase in proliferation, followed by migration to the injured EGL where they initiate expression of *Atoh1* and become GCPs (Fig. 1, A to C) (*12*, *13*, *27*, *28*). Simultaneously, the NEPs in the WM of the lobules reduce their proliferation and differentiation until the EGL is restored, leading to an overall delay in cerebellar development, likely to ensure proper scaling of the different cell types of the cerebellar cortex (*12*). Mosaic mutant analysis revealed that Sonic hedgehog signaling, which normally stimulates proliferation of GCPs and NEPs, is important for regeneration of the cerebellum after irradiation (*12*). However, it is not known what molecular event induces a fate change of BgL-NEPs upon injury and whether a new transitory cellular state facilitates the fate switch.

The commitment of neural stem cells to a neural fate is dependent on a group of proneural basic helix-loop-helix (bHLH) transcription factors (*29*, *30*). In the cerebellum, rhombic lip–derived excitatory neurons and ventricular zone–derived inhibitory neurons are specified by distinct bHLH proteins typified by atonal BHLH transcription factor 1 (ATOH1) and achaete-scute Family BHLH transcription factor 1 (ASCL1), respectively. Genetic inducible fate mapping (GIFM) in the cerebellum indicated that *Ascl1* is transiently expressed sequentially in subsets of ventricular zone– and then NEP-derived cells that generate inhibitory neurons and rarely glial cells and is never involved in GCP production (*25*, *31*). Moreover, ASCL1 is one of the primary proteins found to transform nonneural cells into neurons in vitro. For example, misexpression of *Ascl1* along with *Brn2* and *Myt1l* converts fibroblasts into functional neurons, and ASCL1 acts as a pioneer factor that initiates the reprograming of a fate change via inducing a neurogenic transcriptional program (*32*, *33*). In vivo, ectopic expression of ASCL1 or other ventricular zone–bHLH proteins is able to reprogram astrocytes to form neural cells upon injury (*34*–*38*). In contrast to the required misexpression of ventricular zone–bHLH proteins in all these studies, after cerebellar injury, the fate switch of BgL-NEPs to GCPs does not require forced expression of any genes, highlighting their high plasticity compared to other injury-responsive glial cells outside the cerebellum. However, whether and which proneural bHLH genes play a role in the acquisition of a neural fate by BgL-NEPs during GCP regeneration is unknown.

We used single-cell RNA sequencing (scRNA-seq) in combination with GIFM and loss-of-function studies to define molecularly distinct NEP populations in the postnatal developing cerebellum and uncover a transcription factor involved in the fate switch of a ventricular zone–derived gliogenic BgL-NEP subtype to rhombic lip–derived GCPs. We show that the lobule NEPs consist of two major subtypes, *Hopx*-expressing gliogenic- and *Ascl1*-expressing neurogenic NEPs at steady state and upon injury, and are further subdivided into subpopulations based on the type of astroglia (Bg versus astrocytes) that they will generate, their location (BgL or WM for *Hopx*-NEPs), or the differentiation status of INs (*Ascl1*-NEP lineage). GIFM analysis showed that, whereas at P5 the lineages of the gliogenic and the neurogenic NEPs are mostly exclusive, at P0 a bipotent progenitor exists in the WM. We identified a new transitory cellular state in the BgL upon injury. *Hopx*-expressing NEPs transiently express a transcriptional program that includes *Ascl1* before their migration to the site of injury to replenish the lost GCPs. Conditional mutagenesis showed that *Ascl1* not only is a marker of the transitory cell state but also plays a crucial role in GCP regeneration via suppressing Bg differentiation and facilitating adaptive reprograming. Collectively, our results reveal the molecular diversity and the cellular plasticity of cerebellar NEPs and identify a context- (injury-)dependent transitory cellular state responsible for a glial-to-neural fate switch that enables neonatal cerebellar regeneration.

## RESULTS

### A scRNA-seq approach to identify NEPs during homeostasis and repair

To identify molecularly distinct NEP subtypes that are present in the postnatal cerebellum and new cellular states responsible for their adaptive reprogramming after injury to replenish GCPs, we performed scRNA-seq on dissociated cells from four male P5 *Nes-Cfp/*+ pups that were irradiated (IR) at P1 or control littermates that were not irradiated (nonIR) in two replicate experiments (Fig. 1, A to D, and fig. S1, A to C). P5 is a stage when NEPs are generating INs, astrocytes, and Bg during homeostasis and, in IR, mice NEPs are undergoing adaptive reprogramming. An automated microwell array–based platform (*39*) was used to profile a total of ~19,000 single cells, followed by subclustering to identify pervasive and specific markers for molecularly distinct cell types during homeostasis and upon injury. Clustering of cells (11,933 nonIR and 6996 IR; Fig. 1, E and F, and fig. S1, D to F) using Phenograph (*40*) revealed the expected variety of cerebellar cell types with the greatest number being neurons/progenitors of which the majority were GCPs and granule cells based on the expression of *Atoh1* (GCP) and *Barhl1* (GCP/granule cells) (Fig. 1, E and F; fig. S1, D to F; and table S1). The other major cell types present included Bg and astrocytes and their progenitors (referred to as astroglia; fig. S1, D and F), oligodendrocyte progenitors/oligodendrocytes, Purkinje cells, microglia, and parenchymal cells (fig. S1, D and F). We used *Cfp* transcripts as a surrogate to identify clusters containing NEPs and found that *Cfp* expression was enriched [FDR (false discovery rate) < 0.05] in two clusters with astroglia (C6) and one with ventricular zone–derived neural progenitors (based on *Ascl1 and Ptf1a* expression, C9), as well as in minor clusters with oligodendrocyte progenitors (C7 and C11), ependymal cells (C15), and endothelial/perivascular cells (C16) (Fig. 1, E and F; fig. S1, D and F; and table S1). *Cfp*-expressing cells also were scattered throughout the neural clusters (Fig. 1, E and F). To further computationally analyze the majority of NEPs, we defined NEPs as all the cells in the astroglial and ventricular zone–derived neural progenitor clusters enriched for *Cfp* transcripts (clusters C6 and C9, fold change > 2 and FDR < 0.05) and all of the *Cfp*^+^ cells present in the neural progenitor/neural clusters (C1 to C5) and astrocytes (C8) (Fig. 1, D to F, and fig. S1, A and B). Analysis of *Cfp* transcripts and protein levels, by RNA in situ hybridization and immunofluorescent analysis, respectively, on cerebellar sections from P5 nonIR and IR *Nes-Cfp/*+ cerebella confirmed that the *Cfp* transcript has overlapping expression with cyan fluorescent protein (CFP) protein (as well as SOX2, a pan-NEP/Bg/astrocyte marker) and therefore *Cfp* RNA faithfully identifies NEPs in our scRNA-seq data (fig. S2, A to H). Notably, because our analysis of sections of P5 *Nes-Cfp/*+ cerebella showed that the CFP^+^ cells in the deep WM surrounding the cerebellar nuclei represent <10% of all the CFP^+^ cells in the cerebellum (fig. S2, I to K), these cells are likely poorly represented in our scRNA-seq data.

### scRNA-seq identifies two major subtypes of NEPs in the P5 cerebellum

To identify subpopulations of NEPs, we next clustered the 3391 NEPs as defined above from nonIR (2915 cells) and IR (1376 cells) P5 mice (Fig. 1, G to I, and table S1). Cluster analysis using Phenograph revealed seven NEP clusters (N1 to N7), and several clusters had most of the nonIR or IR cells (Fig. 1, G to L). Analysis of the Allen Brain Atlas RNA in situ mouse P4 dataset for significantly enriched genes in each cluster and lineage markers allowed determination of cluster identities. *Nes-Cfp* and endogenous *Nes* and *Sox2* transcript levels were highest in N1, N6, and N7, suggesting that they include undifferentiated NEPs. The other four clusters (N2 to N5) appeared to be the immediate neural progenitors of NEPs because they had low-level expression of *Sox* and *Nes* and were enriched for genes expressed in neurons (e.g., *Rbfox3* and *Tubb3*) and a gene enriched in the WM (*Igbfbp5*) based on RNA in situ analysis (Allen Brain Atlas; Fig. 1, J, K, and M). All seven clusters showed extensive expression of proliferation markers (*mKi67* and *Top2a*) confirming their progenitor status (Fig. 1K).

Within the undifferentiated NEP clusters, while all three (N1, N6, and N7) expressed the pan-astrocyte genes *Slc1a3* and *Fabp7*, only N1 and N7 expressed *Aqp4* (Fig. 1, J and K). N1 was enriched for Bg lineage genes (*Hopx* and *Gdf10*) (*41*–*43*), indicating that the cells are Bg progenitors. The proneural ventricular zone–bHLH gene *Ascl1* was enriched in N6 (Fig. 1, J, K, and M), indicating that these cells represent the WM-NEPs that are IN progenitors. Other immature IN marker genes such as *Pax2* were not detected at high levels in our dataset. One gene enriched specifically in N7 was *Slc6a11*, an astrocyte-specific γ-aminobutyric acid transporter (*44*), indicating that these NEPs are astrocyte progenitors. However, some cells in cluster N7 expressed neural genes and *Hopx*, raising the possibility that the cluster includes multipotent NEPs that generate INs and astrocytes. Differential expression analysis between the Bg-like NEPs (N1) and neurogenic NEPs (N6) further highlighted the astrogliogenic and neurogenic gene signatures of each NEP subtype (fig. S2L and table S2) and underlines *Hopx* and *Ascl1* as markers of the gliogenic- and neurogenic-NEP subtypes, respectively. Last, immunofluorescent analysis of HOPX and ASCL1 on sections from P5 nonIR *Nes-Cfp/*+ cerebella confirmed that these proteins are coexpressed with CFP (fig. S2, M to R), further highlighting that the cells represent NEP subtypes. Collectively, our scRNA-seq analysis shows that the primary molecular signatures that distinguish NEP populations is based on their lineage propensities as two major NEP subtypes were identified: gliogenic (N1 and N7) and neurogenic (N6) with the gliogenic splitting into astrocyte and Bg lineages. In addition, the more differentiated neuronal progenitors (N2 to N5) split into four groups with different proportions of nonIR and IR cells (Fig. 1L).

### *Ascl1*- and *Hopx*-expressing NEPs have distinct cell lineages

**Fig. 2.**
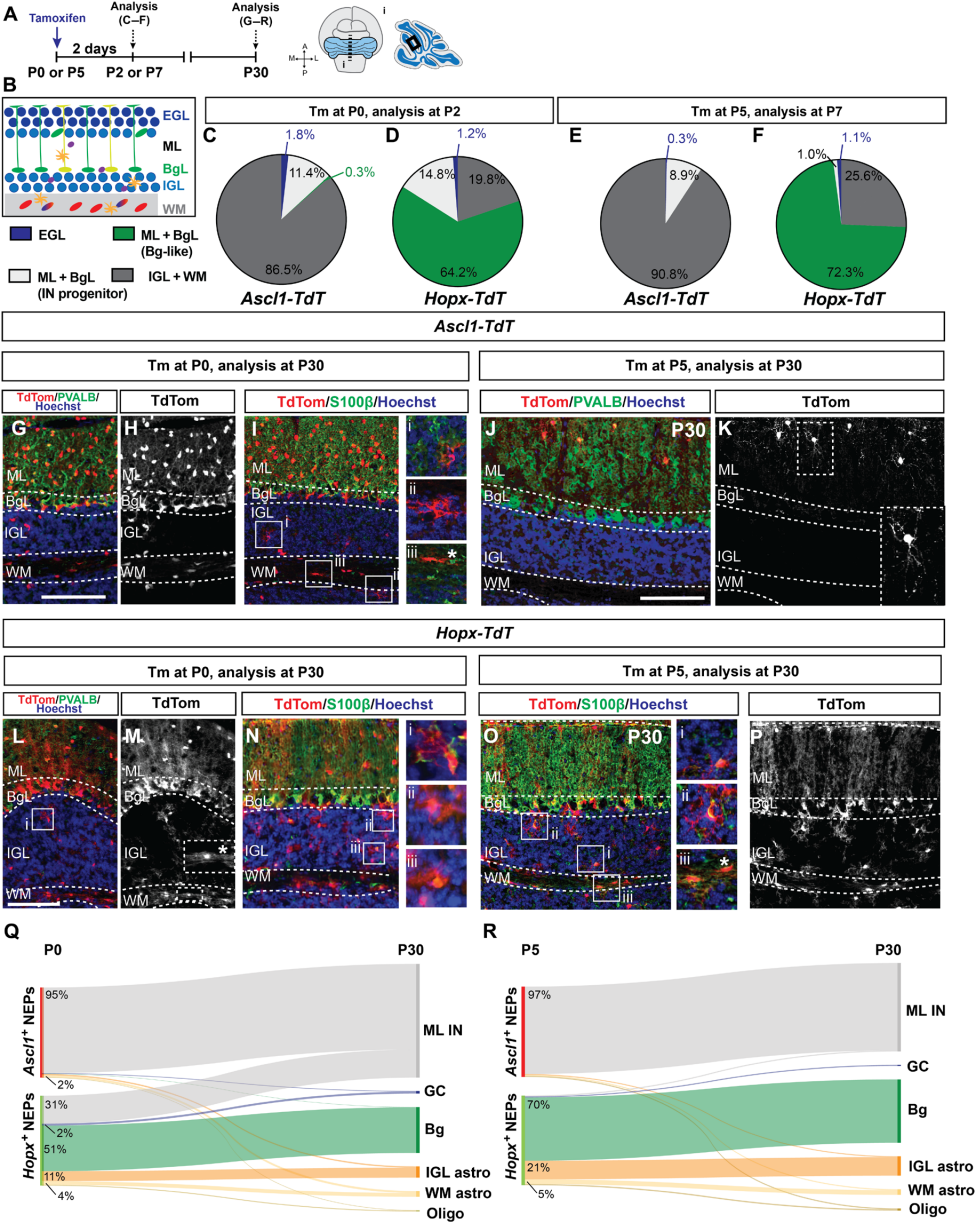
Genetic-induced fate mapping identifies the lineages of the *Ascl1*^+^ neurogenic NEPs and the *Hopx*^+^gliogenic NEPs. (**A** and **B**) Experimental plan and the region of the cerebellum shown in the images. (**C** to **F**) Distribution of the TdT^+^ cells by layer 2 days after Tm administration [(C and D) Tm at P0; (E and F) Tm at P5] in *Ascl1-TdT* [(C) (*n* = 4); (E) (*n* = 3)] and *Hopx-TdT* [(D and F) (*n* = 3)] cerebella. (**G** to **K**) Immunofluorescent (IF) analysis used for the quantification of the TdT^+^ cells by cell type at P30 [(G to I) Tm at P0; (J and K) Tm at P5] in *Ascl1-TdT* animals (*n* = 4 and *n* = 3 brains, Tm at P0 and P5, respectively). Insets in (I) show representative examples of IGL and WM astrocytes and a rare oligodendrocyte (iii, asterisk). Inset in (K) shows a stellate neuron. (**L** to **P**) IF used for analysis and the quantification of the distributions of the TdT^+^ cells by cell type at P30 [(L to N) Tm at P0; (O and P) Tm at P5] in *Hopx-TdT* animals (*n* = 3 brains per age). Insets in (L) to (N) and (O) show representative examples of IGL and WM astrocytes and rare oligodendrocytes (asterisk). (**Q** and **R**) Sankey plots showing the distribution of the progeny of the neurogenic (*Ascl1*^+^) and the gliogenic (*Hopx*^+^) NEPs at P30. Only percent values that are ≥2 are shown in the graphs (refer to tables S3 and S4 for all the values). The overlap between the *Ascl1*^+^ and *Hopx*^+^ cells [~10% at P0 is not represented in (P) for simplicity, see fig. S5]. Scale bars, 50 μm. PVALB, parvalbumin.

To further explore the undifferentiated NEP subtypes identified via scRNA-seq, we determined their lineages by performing GIFM for the neurogenic NEPs using an *Ascl1^CreERT2^* allele and for the two gliogenic-NEP populations using a *Hopx^CreERT2^* allele in combination with a *R26^lox-STOP-loxTdTomato^* reporter (*Ascl1-TdT* and *Hopx-TdT* mice, respectively). We administered tamoxifen (Tm) at P0 and at P5 to assess their progeny at the time of injury and approximately when our scRNA-seq data were collected, respectively (Fig. 2A). Short-term GIFM (analysis 2 days after Tm injection) in *Ascl1-TdT* mice following Tm administration at P5 confirmed that almost all the cells were SOX2^+^ and restricted to the lobule WM with scattered cells in the internal granule layer (IGL) at P7 (90.8 ± 4.0%, *n* = 3; Fig. 2, B and E, and fig. S3, A and E to G). Furthermore, the cells outside the WM had the appearance of migrating IN progenitors destined for the molecular layer (ML) (fig. S3, A and E to G). When Tm was administered instead at P0, the majority of the initially labeled cells in *Ascl1-TdT* mice at P2 were again SOX2^+^ cells mainly restricted to the WM and forming IGL (86.5 ± 0.9, *n* = 4), although there was a small increase in the percentage of labeled cells in the EGL compared to Tm administration at P5 [P0: 1.8 ± 0.9% (*n* = 4) versus P5: 0.3 ± 0.2%, (*n* = 3)] and labeling of very rare Bg-like cells was observed (Fig. 2, B and C, and fig. S3, B to D). When the progeny of the *Ascl1-TdT*^+^ cells were analyzed at P5, 4 days after Tm at P0 to allow differentiation, most of the TdT^+^ cells in the WM and IGL were PAX2^+^ (SOX2 low or negative), and only rare S100β^+^ cells were observed following labeling at P0 (fig. S4, A to G). Thus, at both P0 and P5, *Ascl1-TdT* GIFM labels almost exclusively NEPs in the WM, and labeling with PAX2 indicates that at least some of the progeny will form INs.

Long-term GIFM in *Ascl1-TdT* mice revealed that, in fact, almost all the progeny of the *Ascl1*^+^ NEPs labeled at P0 and P5 are PVALB^+^ ML INs in the outer layer of the cerebellum at P30 (P0: 95.1 ± 2.3%, *n* = 4, P5: 97.0 ± 4.5%, *n* = 3; Fig. 2, G to K, Q, and R, and table S3). As expected, because ML INs are produced in an inside-to-outside manner after birth, the progeny of the *Ascl1*^+^ NEPs labeled at P5 was restricted to the outer ML containing stellate cells (latest-born INs) (Fig. 2, J and K), whereas the progeny of the *Ascl1*^+^ NEPs labeled at P0 included both later-born INs and earlier-born basket neurons in the inner ML that have processes in the BgL (Fig. 2, G and H). Only rare S100β^+^ astrocytes in the lobule WM or IGL and very rare oligodendorcytes and no granule cells were labeled in the P30 cerebellum after labeling at P5 with Tm (Fig. 2R and table S3). More astrocytes were generated from P0 *Ascl1*^+^ NEPs than P5 (primarily in the WM, 2.15 ± 1.1%, *n* = 4 compared to Tm at P5: 1.1 ± 1.6%, *n* = 3), in addition to very rare granule cells and Bg (Fig. 2, I and Q, and table S3). These results not only confirm that there is an *Ascl1*^+^ neurogenic NEP subtype in the lobule WM of the neonatal cerebellum but also demonstrate that it has a restricted lineage as early as P0 to almost exclusively ML INs. Rare glia are also detected with *Ascl1-TdT* GIFM, and the proportion is slightly higher at P0 raising the possibility of a bipotent WM progenitor earlier in development (Fig. 2, Q and R).

In contrast to *Ascl1-TdT* GIFM, short-term labeling in P7 *Hopx-TdT* animals given Tm at P5 showed that the majority of the TdT^+^cells were in the BgL (72.3 ± 6.2%, *n* = 3; Fig. 2F and fig. S2, H and I). Marker analysis showed that all TdT^+^ cells were SOX2^+^ (fig. S3, K and L). In the WM of the lobules, a subpopulation of SOX2^+^ cells was TdT^+^ (25.6 ± 6.1% of TdT^+^ cells, *n* = 3) along with very rare SOX2^+^ cells in the inner EGL (1.1 ± 0.3% of TdT^+^ cells, *n* = 3, Fig. 2, B and F, and fig. S3, K to M). In contrast, when Tm was administered at P0, short-term labeling revealed that while all TdT^+^ cells were SOX2^+^ and the majority were in the BgL (64.2 ± 3.1%, *n* = 3), there was an increase in the proportion of TdT^+^ cells that were in the IGL and WM (19.8 ± 4.3%, *n* = 3) or appeared to be migrating IN progenitors in the ML + BgL because they lacked a Bg-like morphology (14.8 ± 1.2%, *n* = 3)(Fig. 2, B and D, and fig. S3, H to J). When the progeny of the *Hopx-TdT^+^* cells were analyzed at P5, 4 days after Tm at P0 to allow labeled cells to differentiate, we observed that most of the TdT^+^ cells in all layers were S100β^+^, but in addition rare PAX2^+^ *Hopx-TdT^+^* cells were detected (fig. S4, H to M). These results indicate that *Hopx*-expressing NEPs in the WM at P0 give rise to INs in addition to astrocytes and thus could be bipotent (Fig. 2, B, D, and F, and figs. S3, H to M, and S4, H to M).

Long-term GIFM of *Hopx-TdT* mice at P30 showed that when Tm was administered at P5, 98.2 ± 0.6% (*n* = 3) of the TdT^+^ cells were astroglia (70.4 ± 2.7% Bg, 25.8 ± 2.1% astrocytes in the IGL and WM) and rare oligodendrocytes in the WM (1.3 ± 1.0%) and only ~1.8% were inhibitory neurons (1.1 ± 0.3% in the ML) (Fig. 2, O, P, and R, and table S4). In contrast, 31.1 ± 1.3% of the progeny of the *Hopx*-expressing NEPs labeled at P0 were ML INs at P30 (*n* = 3; Fig. 2, L to N and Q). Together, our GIFM results demonstrate that *Hopx*^+^ WM-NEPs give rise to both ML INs and astrocytes, providing evidence for a bipotent NEP progenitor at P0 in the WM (Fig. 2, Q and R, and table S4). Most of the *Hopx*-NEPs, however, give rise to Bg and/or astrocytes in the lobules.

Proliferation analysis of both (*Ascl1*^+^) and (*Hopx^+^)* short-term fate mapped NEPs using 1-hour 5-ethynyl-2′-deoxyuridine (EdU) incorporation showed that all NEP populations are proliferative, confirming their progenitor status (fig. S3, N to O). Last, both *Hopx-TdT* and *Ascl1-TdT* cerebella that were administered Tm at P0 and analyzed at P2 for ASCL1 or HOPX protein, respectively, showed that ~10% of the TdT^+^ cells were double labeling, revealing that a subset of NEPs in the WM express *Ascl1* and *Hopx*. Thus, there is an overlap in the two progenitor populations being labeled with each CreER line at P0, whereas at P5 the overlap was less than 1% (fig. S5).

RNA in situ analysis of the WM-NEP marker *Igfbp5* showed expression in the WM of both the deep cerebellum and lobules, whereas *Hopx* and *Ascl1* expression was restricted to the lobules at P5 (fig. S6, C to E). Consistent with the expression, there was no labeling in the deep WM cells at both P7 and P30 after Tm was administered at P5 both in the *Ascl1-TdT* and the *Hopx-TdT* cerebella (fig. S6, F to M). This result reveals that the lineages of NEPs expressing these genes at P5 are restricted to the lobules. Unexpectedly, when Tm was administered at P0, *Ascl1-TdT* GIFM but not *Hopx-TdT* GIFM showed labeling of cells at P2 and P30 in the deep WM. *Ascl1-TdT^+^* cells were all SOX2^+^ at P2, and the cells had glial morphology and were mostly S100β^+^ at P30 (fig. S6, N to U, and table S5). Given that the INs of the cerebellar nuclei are generated during embryonic development directly from the ventricular zone, the deep WM-NEPs should only be gliogenic postnatally. However, a specific marker for this population of NEPs is yet to be identified.

Overall, the GIFM studies validate the identities of the astrogliogenic (*Hopx*^+^)– and neurogenic (*Ascl1*^+^)–NEP subtypes at P5 obtained via scRNA-seq (Fig. 1F) and also uncovered a WM-NEP population in the lobules that expresses *Hopx* and primarily produces astrocytes at P5 but also generates ML INs at P0 (Fig. 2, Q and R). GIFM also demonstrates that *Ascl1*-expressing NEPs have a lineage that is largely restricted to ML INs at P0 and P5 (Fig. 2, Q and R). Furthermore, WM-NEPs expressing *Ascl1* or *Hopx*, especially *Hopx*^+^, appear to have greater plasticity at P0 than P5, suggesting the existence of a bipotent progenitor that produces astrocytes and ML INs.

### Transcriptional programs of *Ascl1*-NEPs diverge between nonIR and IR mice

**Fig. 3.**
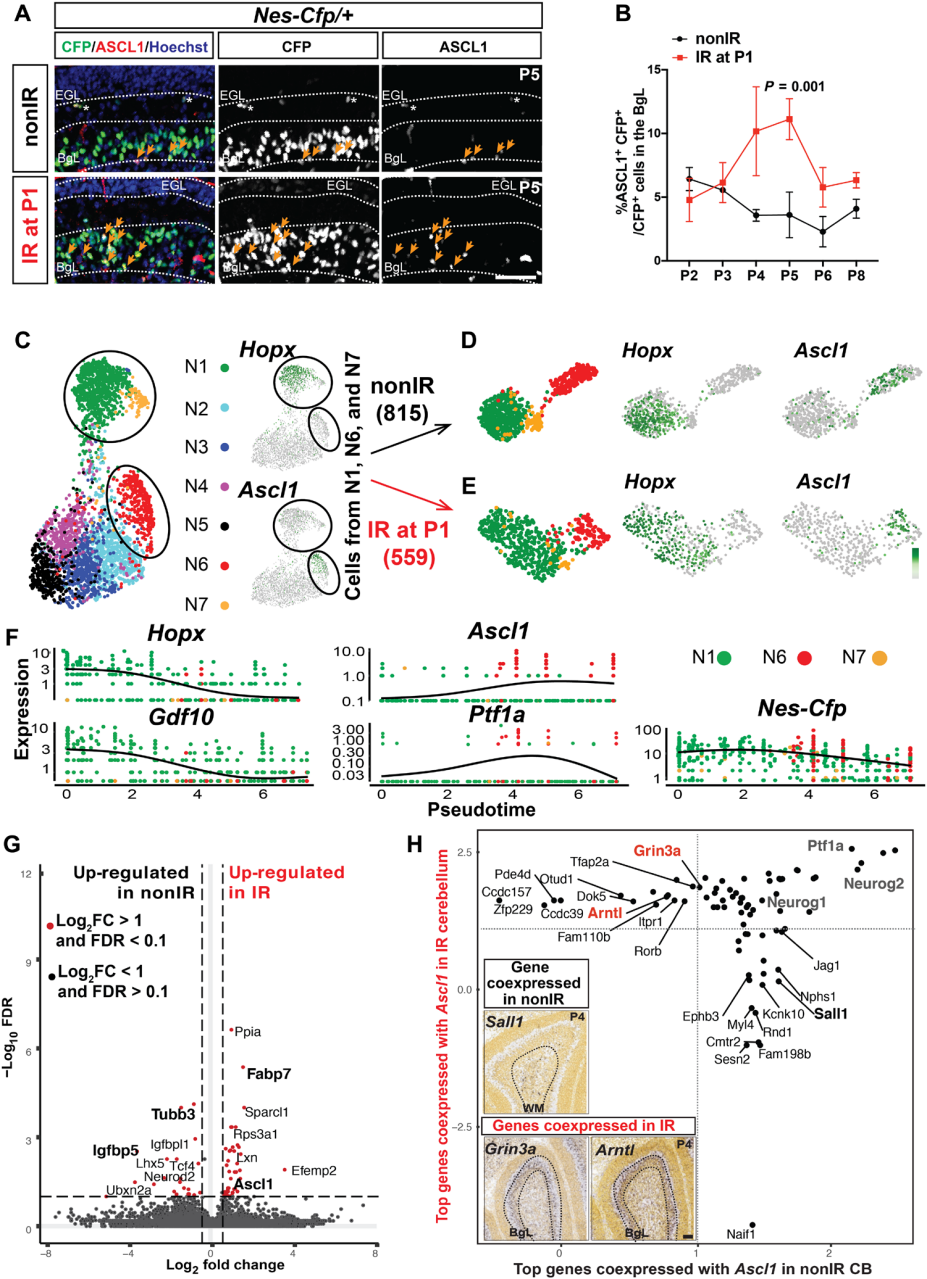
Transcriptional programs of *Ascl1*^+^ NEPs diverge between nonIR and IR conditions and a transitory *Ascl1*^+^ transitory cell state emerges upon injury. (**A**) IF analysis for ASCL1 and CFP on P5 nonIR and IR *Nes-Cfp* cerebella. (**B**) Quantification of percentage of ASCL1^+^ CFP^+^ cells in the BgL at P2 to P6 and P8 in nonIR and IR *Nes-Cfp* cerebella [two-way analysis of variance (ANOVA), *F*_(1,15)_ = 25.75, *P* = 0.0001]. (**C** to **E**) UMAP showing subclustering of the nonIR (B) and the IR (C) cells from clusters N1, N6, and N7 (circled; Fig. 1F). (**F**) Pseudotemporal ordering of the IR NEPs from N1, N6, and N7 using Monocle3. Cells are colored on the basis of their original cluster identity shown in (C). Expression levels of marker genes *Hopx*, *Gdf10* (astroglia), *Ascl1*, *Ptf1a* (neurogenic), and *Nes-Cfp* (pan-NEP) are plotted with respect to pseudotime. (**G**) Volcano plot showing the differentially expressed genes [fold change (FC)] obtained from the comparison of the nonIR cells to the IR cells in cluster N6 (*Ascl1*-enriched NEPs; table S6). (**H**) Analysis of the genes that are most likely to be coexpressed with *Ascl1* in the nonIR and IR NEPs. Quadrants represent whether a gene is coexpressed only in the nonIR cells (lower right) or IR cells (upper left) or in both condition (top right). Insets show Allen Brain Atlas RNA in situ analysis for *Sall1*, *Grin3a*, and *Arntl* in the P4 cerebellum. Scale bar, 50 μm.

We next assessed the effects of injury on the neurogenic and gliogenic NEPs by RNA in situ analysis, in particular to understand how the fate of gliogenic NEPs in the BgL is switched to GCPs upon injury. RNA in situ analysis of the gliogenic NEP marker *Hopx* and Bg lineage marker *Gdf10* on sections from nonIR and IR P5 cerebella showed that their expression pattern was not changed by irradiation, as expression remained in the BgL with weak *Hopx* in the WM. As expected, the BgL was thicker reflecting the increased proliferation of BgL-NEPs upon irradiation (fig. S7, A to D) (*12*). Expression of the WM-NEP marker *Igfbp5* was reduced in IR NEPs in both the lobules and deep cerebellum based on RNA in situ analysis (fig. S7, E to I), consistent with a reduction in the number of *Igfbp5*-expressing cells detected via scRNA-seq (nonIR: 750 of 2015 versus IR: 153 of 1376 cells, and Fig. 1K). Notably, RNA in situ hybridization for *Ascl1* on P5 nonIR and IR cerebellar sections showed a clear appearance of *Ascl1* expression in the BgL after irradiation (fig. S7, J and K). To confirm that injury induces ventricular zone–bHLH gene expression in BgL-NEPs, we performed immunoflourescence analysis of P5 *Nes-Cfp/*+ cerebella and detected a significant increase in the number of ASCL1^+^ CFP^+^ cells in the BgL of IR cerebella compared to nonIR P5 littermates (3.1 ± 0.4–fold, *n* = 3, *P* = 0.006, Student’s *t* test; Fig. 3, A and B, and fig. S8, A to F). Moreover, quantification of the number of ASCL1^+^ CFP^+^ cells every day between P2 and P6 and at P8 showed that expression in BgL-NEPs peaks 3 to 4 days after irradiation (Fig. 3B). In summary, RNA in situ hybridization and immunoflourescence analysis revealed that after injury, *Igfbp5* expression is reduced throughout the WM, whereas the *Ascl1* becomes ectopically expressed in the BgL.

To assess the effects of injury on the transcriptional programs of the *Hopx*^+^ (N1) and *Ascl1*^+^ (N6) NEPs and the neural progenitors (clusters N2 to N5), we performed gene set enrichment analysis (GSEA) of the differentially expressed genes between nonIR and IR cells obtained from the scRNA-seq. In the *Hopx*-enriched cluster (N1; fig. S7L and table S6), we found an increase in Gene Ontology terms for antioxidant activity and response to oxygen radicals, whereas for the *Ascl1*-enriched cluster (N6; fig. S7M and table S6), cell fate determination, regulation of stem cell activity, and astrocyte differentiation were increased upon injury. In the NEPs from clusters N2 to N5, we observed up-regulation of the Gene Ontology terms for metabolic processes and cell cycle regulation in IR cells whereas terms related to neural differentiation and activity were increased in nonIR cells (fig. S7N and table S6), suggesting that the main differences between the IR and nonIR neural progenitors are related to their differentiation status. Thus, injury induces distinct transcriptional changes in the NEP subtypes and subpopulations.

To further assess the injury-induced changes on the gliogenic (N1 and N7) and *Ascl1*^+^ neurogenic (N6) NEPs, we clustered only the nonIR (*n* = 815) or IR (*n* = 559) cells from the three clusters (Fig. 3, C to E). When nonIR cells from N1, N6, and N7 were subclustered, gliogenic NEPs (*Hopx^+^*) and neurogenic NEPs (*Ascl1*^+^) formed two well-separated clusters in the Uniform Manifold Approximation and Projection (UMAP) space (Fig. 3D), in line with the GIFM analysis at P5 showing that the NEP subtypes generate distinct cell types (Fig. 2R). When the IR cells from N1, N6, and N7 were subclustered, gliogenic and neurogenic NEPs showed more of a continuum in the UMAP space (Fig. 3E). Pseudotime analysis on the IR NEPs from clusters N1, N6, and N7 (Fig. 3E) using Monocle3 further confirmed that IR *Hopx*-expressing cells precede IR *Ascl1*-expressing cells and showed that the expression of BgL-NEP genes (*Hopx* and *Gdf10*) were diminished as the *Ascl1* (and *Ptf1a*) expression increased, while overall, *Nes-Cfp* expression remained high throughout (Fig. 3F). Furthermore, differential expression analysis of the nonIR cells compared to the IR cells in neurogenic cluster N6 showed up-regulation of genes such as *Fabp7*, which are seen in astroglia as well as *Ascl1* itself in IR cells (log_2_ fold change > 1, FDR < 0.1; Fig. 3G and table S7), whereas in the nonIR condition, genes differentially up-regulated included neural genes (*Tubb3*) or WM genes such as *Igfbp5* (log_2_ fold change > 1, FDR < 0.1; Fig. 3G and table S7). These findings indicate that a transitional state arises after injury with *Ascl1* as a marker gene, and together with the RNA in situ analysis, we propose that, upon injury, BgL-NEPs transition into a new neurogenic state typified by *Ascl1* expression.

To further test this idea, we assessed coexpression and mutual exclusivity for all gene pairs and calculated probability ratios of detecting two genes in the same cell (*45*) for the top 50 genes with high *Ascl1* coexpression probability ratios (18 of 100 showed high coexpression probability ratios with *Ascl1* in both IR and nonIR cells; table S8). Among the genes coexpressed with *Ascl1* in both conditions were three other proneural ventricular zone–bHLH genes, *Ptf1a* and *Neurog1/2* (Fig. 3H). We also detected sets of genes that were coexpressed with *Ascl1* only in nonIR or in IR NEPs (Fig. 3H). Analysis of P4 RNA in situ data (Allen Brain Atlas) for *Sall1* that is coexpressed with *Ascl1* in nonIR NEPs revealed that its expression is restricted to the WM where *Ascl1*^+^ NEPs normally reside (Fig. 3H). *Grin3a* and *Arntl* that are coexpressed with *Ascl1* in IR NEPs are expressed in the BgL, consistent with the idea that after irradiation *Ascl1* is induced in some BgL-NEPs (Fig. 3H). Furthermore, *Grin3a* and *Arntl* have been implicated in astrocyte injury-related functions (*46*, *47*). RNA in situ hybridization analysis for *Grin3a* and *Arntl* on P5 nonIR and IR cerebellar sections confirmed their BgL expression pattern and showed no ectopic expression upon irradiation (fig. S8, G to J). Our scRNA-seq comparison of P5 nonIR and IR NEPs and in vivo identification of the location of the NEP subtypes provide evidence that there is a new transitory state in the BgL-NEPs after irradiation with a mixed glial and neurogenic gene signature and that an injury-responsive gliogenic BgL-NEP population turns on *Ascl1* upon injury.

### Granule cells are derived from *Hopx*-NEPs after injury

**Fig. 4.**
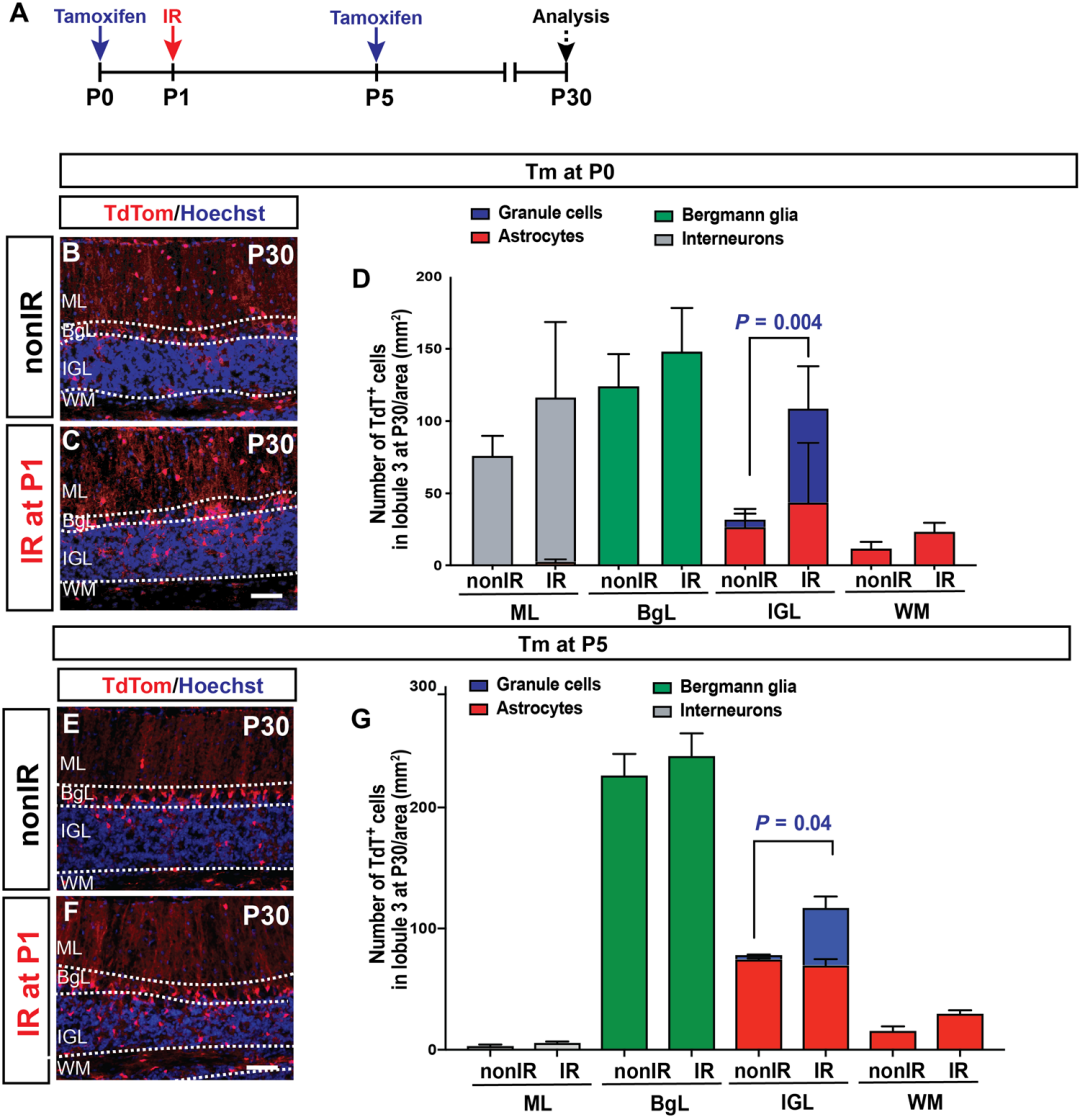
*Hopx*-NEPs give rise to granule neurons. (**A**) Experimental plan. (**B** and **C**) IF analysis of P30 nonIR and IR *Hopx-TdT* brains that were given Tm at P0. (**D**) Quantification of the density of the progeny of nonIR and IR *Hopx-TdT* brains at P30 (Tm at P0) by cell types and the layer of the cerebellum (*n* = 3 per condition, two-way ANOVA, *F*_(7,42)_ = 8.718, *P* < 0.0001). (**E** and **F**) IF analysis of P30 nonIR and IR *Hopx-TdT* brains that were given Tm at P5. (**G**) Quantification of the density of the progeny of nonIR and IR *Hopx-TdT* brains at P30 (Tm at P5) by cell types and the layer of the cerebellum (*n* = 3 per condition, two-way ANOVA, *F*_(1,14)_ = 5.047, *P* = 0.04). Scale bars, 50 μm.

To demonstrate that *Hopx-*derived BgL-NEPs give rise to GCPs and granule cells after irradiation, we performed GIFM with *Hopx-TdT* animals (Fig. 4A and fig. S9A). Following Tm administration at P0 (Fig. 4, B to D) or P5 (Fig. 4, E to G) and analysis at P30, we observed a significant increase in the density of TdT^+^ granule cells in the IGL of IR *Hopx-TdT* brains compared to their nonIR littermates (Fig. 4, D and G, D: two-way analysis of variance (ANOVA), *F*_(7,42)_ = 8.718, *P* < 0.0001, *n* = 3 brains per condition; G: *F*_(1,14)_ = 5.047, *P* = 0.04, *n* = 3 brains per condition). At P7, TdT^+^ cells were present in the EGL of the IR cerebella in the *Hopx-TdT* pups that were given Tm at P0 or at P5, and most expressed SOX2, indicating that progeny of SOX2^+^ *Hopx-TdT^+^*–labeled BgL-NEPs migrated to the site of injury (fig. S9, B to K). Last, as during homeostasis, no TdT^+^ cells were detected in the deep WM of IR *Hopx-TdT* animals that were given Tm at P0 or at P5 (fig. S9L). Overall, these results provide experimental evidence that both P0 and P5 *Hopx*^+^ BgL-NEPs change their fate and become GCPs and then granule cells upon injury.

### ASCL1 marks a neurogenic BgL-NEP population during adaptive reprogramming that gives rise to GCs

**Fig. 5.**
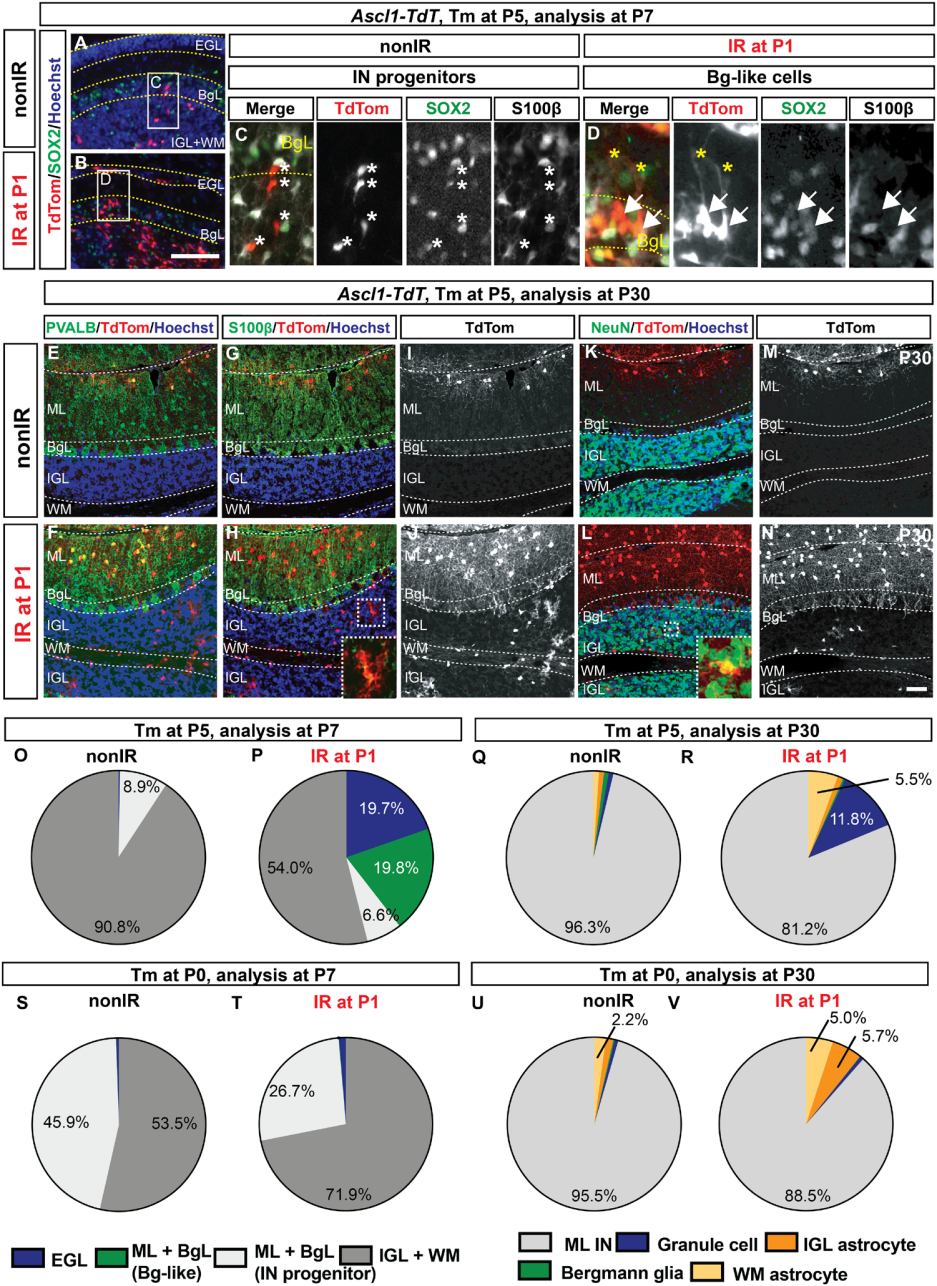
Injury-induced *Ascl1*^+^ BgL-NEPs give rise to granule cells and astrocytes but not Bg. (**A** and **B**) IF analysis of *Ascl1-TdT* brains at P7 (48 hours after Tm). (**C** and **D**) Insets show a migrating IN progenitors [asterisks; (C)] and a SOX2^+^ S100β^−^ BgL-like *Ascl1-TdT* cell [arrows; (D)]. Yellow asterisks show the pial projection of the TdT^+^ cells. (**E** to **N**) IF analysis of nonIR and IR P30 *Ascl1-TdT* cerebellum that was given Tm at P5 showing examples of the TdT^+^ ML INs (PVALB), astrocytes (S100β), and granule cells (NeuN in the IGL). (**O** to **V**) Proportions of TdT^+^ cells at P7 (O. P, S, and T) or at P30 (Q, R, U, and V) from nonIR (O, Q, S, and U) and IR (P, R, T, and V) *Ascl1-TdT* cerebella that are given Tm at P5 [(O to R), *n* ≥ 3 brains per condition] or at P0 [(S to V) *n* = 3 brains per condition]. Only percent values that are ≥2 are shown in the graphs (refer to table S9 for all the values). Scale bars, 100 μm (B to G) and 50 μm (K to T).

To confirm that *Ascl1* marks a new BgL-NEP transitory cellular state after irradiation that generates GCPs and granule cells, we performed GIFM in *Ascl1-TdT* nonIR and IR animals given Tm at P5, the stage when the largest number of injury-induced ASCL1^+^ cells was detected in the BgL (Fig. 3, A and B). As predicted, analysis of P6 and P7 cerebella revealed a population of TdT^+^ BgL-NEPs that are SOX2^+^/S100β^−^ and have glial fibers projecting to the pial surface only in pups irradiated at P1 (Fig. 5, A to D, and fig. S10, A to E). In contrast, the few TdT^+^ cells in the BgL of nonIR cerebella had a morphology of migrating IN progenitors (Fig. 5C and fig. S10D). In addition, the densities of TdT^+^ cells in the EGL and BgL were significantly increased after irradiation (Fig. 5, A, B, O, and P, and fig. S10, B, C, and F). Furthermore, the number of labeled cells in the EGL increased significantly between 24 and 48 hours after Tm at P5 only in IR cerebella (fig. S10F). Analysis of EdU labeling following a 1-hour pulse at P6 demonstrated that the TdT^+^ cells in the EGL and BgL proliferate after irradiation (fig. S10, G and H). Last, live imaging of lobule 3 in thick cerebellar sections from P7 mice given Tm at P5 showed that the *Ascl1-TdT^+^* cells do migrate from the BgL to the EGL in IR cerebella, whereas in nonIR P7 slices, no cells migrate from BgL to EGL (fig. S11, A to D, and movies S1 and S2). These results provide strong evidence that BgL-NEPs express *Ascl1* during adaptive reprogramming and migrate to the EGL, although we cannot exclude the possibility that rare cells in the EGL express *Ascl1-CreER* after injury and contribute a minor component of the regeneration.

Analysis of P30 IR *Ascl1-TdT* cerebella that were administered Tm at P5 revealed several key findings. First, the P30 IR brains had a significant and large increase in the density and the proportion of the TdT^+^ cells that were granule cells (11.8 ± 2.0% of all TdT^+^ cells, *n* = 4 IR compared to 1.0 ± 1.4% of TdT^+^ cells, *n* = 5 nonIR; Fig. 5, E to N, Q, and R; fig. S12, A and B; and table S9). Second, almost no TdT^+^ Bg were detected in the P30 IR *Ascl1-TdT* cerebella, suggesting that *Ascl1* induces the BgL-NEPs to switch from making Bg to GCPs and that the switch is irreversible (Fig. 5, Q and R, and fig. S12, A and B). Third, there was a delay in production of ML INs after irradiation, as demonstrated by an increase in the density of TdT^+^ ML INs per lobule that included both earlier-born basket and later-born stellate cells (Fig. 5, Q and R, and fig. S12, A, B, and E to G). Last, we observed an unexpected increase in lobule astrocyte production in the IR *Ascl1-TdT* brains, primarily in the WM (Fig. 5, Q and R, and fig. S12, A and B). We detected an emergence of TdT^+^ astrocytes at P30 in the deep WM of IR *Ascl1-TdT* cerebella labeled at P5 not seen in nonIR mice (fig. S12H and table S5). However, because no TdT^+^ cells were detected in the deep WM of IR *Ascl1-TdT* cerebella at P7, one possibility is that the TdT^+^ cells originate from the lobule WM, indicating a fate switch including a new migration path (fig. S12H and table S5).

If the *Ascl1*^+^ BgL-NEPs represent an injury induced cellular state, and if Tm is administered at P0 to *Ascl1-TdT* animals, then there should be no increase in labeled GCPs or cells in the BgL at P7 or granule cells at P30 compared to nonIR. As predicted, analysis at P7 showed that, upon irradiation, there were no changes in the numbers or proportions of rare TdT^+^ Bg-like cells or cells in the EGL in IR mice compared to nonIR (Fig. 5, S and T). Consistent with this, at P30, no difference in the labeling of rare granule cells or Bg between IR and nonIR mice was observed (Fig. 5, U and V, and table S9), confirming that the *Ascl1*^+^ neurogenic BgL-NEP is an injury-induced cellular state. P0-labeled *Ascl1*^+^ NEPs in IR mice showed an increase in labeled cells in the IGL and WM at P7 and increase in production of astrocytes in the IGL and WM at P30 compared to nonIR littermates (Fig. 5, S to V, and table S9). Collectively, these results demonstrate that GCPs are generated from an *Ascl1*-expressing BgL-NEP upon injury. Furthermore, irradiation leads to a delay in production of INs and an increase in astrocyte production, likely from normally neurogenic *Ascl1*^+^ WM-NEPs.

### *Hopx*-NEPs give rise to the injury-induced ASCL1^+^ transitory cellular state

**Fig. 6.**
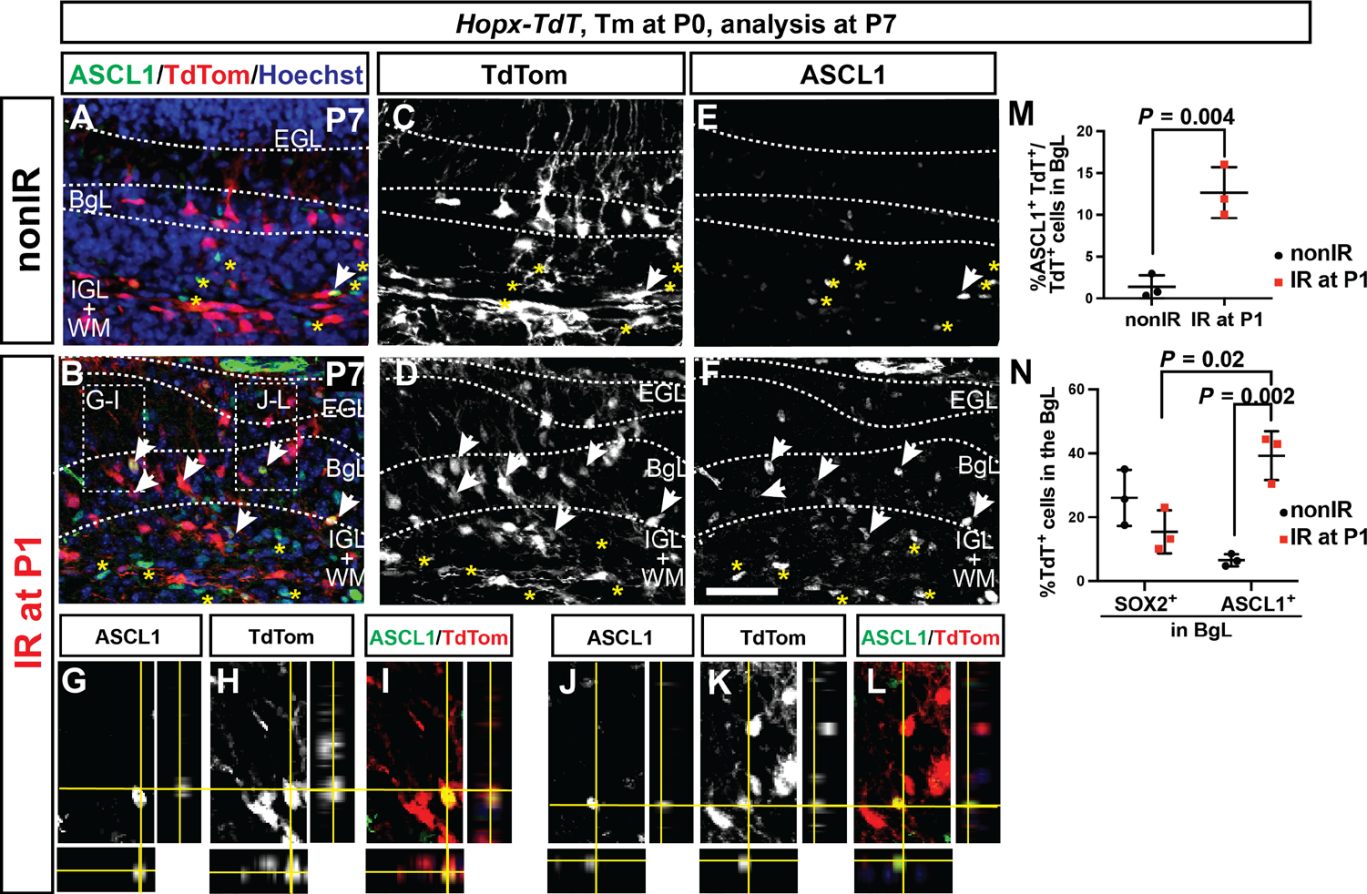
*Hopx*-derived NEPs in the BgL turn on ASCL1 upon injury. (**A** to **M**) IF analysis (A to F) and quantification (M) of the percentage of the TdT^+^ and ASCL1^+^ cells per TdT^+^ cell in the BgL at P7 on sections from *Hopx-TdT* nonIR (A, C, and E) and IR (B, D, F, and G to L) brains (*n* = 3 brains, Student’s *t* test, *P* = 0.004). (G) to (L) are taken from the region indicated in (B) and show orthogonal views demonstrating colocalization of TdT^+^ and ASCL1^+^ in the BgL in IR *Hopx-TdT* cerebella. (**N**) Quantification of the percentage of *Hopx*-derived TdT^+^ cells in all BgL-NEPs and Bg (SOX2^+^ cells) or in all *Ascl1*^+^ BgL-NEPs (*n* = 3 per condition, two-way ANOVA, *F*_(1,8)_ = 7.928, *P* = 0.02). Scale bar, 50 μm.

Our GIFM in *Hopx-TdT* animals given Tm at P0 showed that, unlike *Ascl1*^+^ cells, *Hopx*^+^ cells give rise to the granule cell lineage. We therefore fate-mapped *Hopx*^+^ cells at P0 and asked whether they give rise to ASCL1^+^ cells in the BgL at P7, in addition to GCPs. We observed a significant increase in the percentage of ASCL1^+^ TdT^+^ cells of all TdT^+^ cells in the BgL in IR pups at P7 (12.7 ± 3.1%, *n* = 3) compared to nonIR littermates (1.4 ± 1.4%, *n* = 3; Fig. 6, A to M). In IR pups, the percentage of the ASCL1^+^ TdT^+^ double-positive cells among all the ASCL1^+^ cells in the BgL was higher than the percentage of SOX2^+^ TdT^+^ double-positive cells among all the SOX2^+^ cells (a pan-NEP/Bg/astrocyte marker) in the BgL (39.2 ± 7.7% in IR and 6.5 ± 1.9% in nonIR versus 15.5 ± 6.7% IR and 26.1 ± 8.8% in nonIR, respectively, *n* = 3; Fig. 6N). This result suggests that *Hopx^CreERT2^* preferentially marks the BgL-NEPs at P0 with the ability to undergo adaptive reprograming in response to loss of GCPs at P1.

### *Ascl1* is required in *Hopx*-NEPs for adaptive reprogramming and production of granule cells

**Fig. 7.**
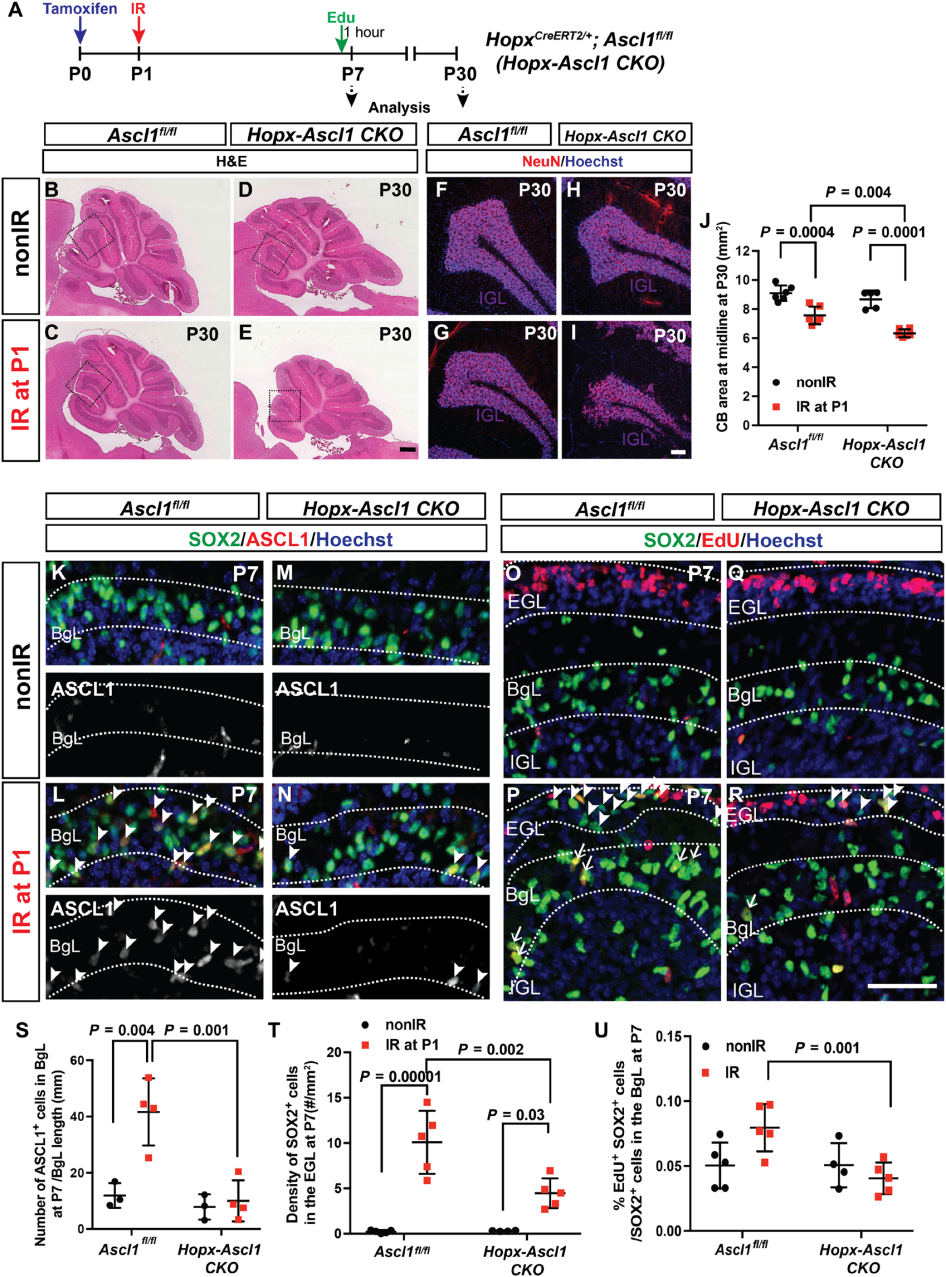
*Ascl1* is required for the adaptive reprograming of BgL-NEPs into granule cells. (**A**) Experimental plan. (**B** to **E**) Hematoxylin and eosin images of midsagittal sections from P30 nonIR and IR control or *Hopx-Ascl1* CKO cerebella. (**F** to **I**) IF analysis of NeuN at P30. (**J**) Quantification of midline cerebellar area (*n* ≥ 5 per condition, two-way ANOVA, *F*_(1,19)_ = 14.39, *P* < 0.001). (**K** to **N**) IF analysis for ASCL1 on sections from P7 nonIR and IR control and *Hopx-Ascl1* cerebella (arrowheads, ASCL1^+^ BgL-NEPs). (**O** to **R**) IF analysis of SOX2 and EdU on sections from P7 nonIR and IR control and *Hopx-Ascl1* CKO cerebella. (Arrowheads show the SOX2^+^ cells in the EGL and arrows show the EdU^+^ SOX2^+^ cells in the BgL). (**S** to **U**) Quantification of the density of ASCL1^+^ cells in the BgL [(S) *n* ≥ 3 per condition, two-way ANOVA, *F*_(1,10)_ = 16.41, *P* = 0.002], density of SOX2^+^ cells in the EGL [(T) *n* ≥ 4 per condition, two-way ANOVA, *F*_(1,15)_ = 58.49, *P* < 0.0001] and percentage of EdU^+^ SOX2^+^ cells within the SOX2^+^ cells in the BgL [(U), *n* ≥ 4 per condition, two-way ANOVA, *F*_(1,15)_ = 6.549, *P* = 0.02]. Scale bars, 500 μm (B to E), 100 μm (F to I), and 50 μm (K to R).

Given the unexpected up-regulation of *Ascl1* in the normally gliogenic BgL-NEPs upon death of GCPs, we tested whether *Ascl1* is required for repair of the granule cell lineage after cerebellar injury using a conditional knockout (CKO) approach. *Hopx^CreERT2/+^; Ascl1^fl/fl^* animals injected with Tm at P0 (*Hopx*-*Ascl1* CKOs) were used to delete *Ascl1* primarily from BgL-NEPs, and their regenerative capacity was compared to *Ascl1^fl/fl^* littermate controls after irradiation at P1 (Fig. 7A). Analysis of P30 cerebella showed a significantly greater reduction in cerebellar area at the midline in IR *Hopx*-*Ascl1* CKOs compared IR littermate controls (Fig. 7, B to E and J). Furthermore, the IGL was less organized in some lobules of the IR mutants than in IR controls, further demonstrating impaired regeneration (Fig. 7, F to I). The loss of one copy of *Ascl1* resulted in a mild but significant reduction in the area of the cerebellum of *Ascl1-TdT* animals compared to *R26^TdT/+^* littermate controls, further confirming that *Ascl1* is required for regeneration after irradiation (fig. S13, E to H and J). To rule out that the impairment in regeneration is due to a loss of one copy of the *Hopx* gene in the *Hopx*-*Ascl1* CKO animals, we compared cerebellar areas of IR and nonIR *Hopx-TdT* animals and their *R26^TdT/+^* littermates and found no significant differences between the genotypes in each condition, showing that loss of one copy of *Hopx* does not impair regeneration (fig. S13, A to D and I).

We next asked whether the number of Bg or astrocytes generated was altered in P30 *Hopx*-*Ascl1* CKOs. There was a mild but significant increase in the density of Bg in IR *Hopx*-*Ascl1* CKOs compared to IR controls, but no differences between the other three groups. One possibility is that, after irradiation, the mutant BgL-NEPs generate Bg rather than GCPs (fig. S13, K to P). In addition, the density of the astrocytes in the lobule WM of the IR *Hopx*-*Ascl1* CKOs was increased compared to nonIR mutants and IR controls (fig. S13, Q and R). Last, we quantified the density of ML INs because we observed a population labeled with *Hopx^CreERT2^* upon Tm administration at P0. No significant change in the density of ML INs was observed after irradiation in the *Hopx*-*Ascl1* CKOs compared to their control littermates or with their respective nonIR controls (fig. S13, S and T). However, because the size of the mutant cerebellum is significantly reduced, by extrapolation, the total number of ML INs is reduced after irradiation in mutants.

To understand why the regeneration is impaired in the *Hopx-Ascl1* CKOs, we analyzed nonIR and IR cerebella at P7 and determined whether proliferation of NEPs and/or their migration to the EGL were affected. Quantification of the density of ASCL1^+^ cells in the BgL in P7 cerebella confirmed loss of ASCL1-expressing transitory cells after irradiation and thus deletion of *Ascl1* in mutants (Fig. 7, K to N and S). Significantly, *Hopx*-*Ascl1* CKOs had a lower density of SOX2^+^ cells in the EGL after injury (BgL-derived cells that had migrated to the injury) compared to their control IR littermates (Fig. 7, O to R and T). A previous study suggested that proliferation genes are direct targets of ASCL1 in embryonic stem cell–derived neural stem cell cultures or the embryonic ventral brain (*48*). To test whether loss of *Ascl1* in the *Hopx*-expressing NEPs impairs their proliferation after injury, we injected EdU 1 hour before euthanizing P7 pups (Fig. 7A) and assessed the percentage of EdU^+^ SOX2^+^ cells of all SOX2^+^ cells in the BgL. A significant decrease in the percentage of EdU^+^ SOX2^+^ cells was detected in the BgL of IR *Hopx*-*Ascl1* CKOs compared to controls (Fig. 7, O to R and U). In summary, these results show that ASCL1 is required for the full reprograming of *Hopx*-expressing BgL-NEPs after depletion of GCPs, including efficient proliferation and migration to the EGL, and as a consequence of impaired replenishment of GCPs, the cerebella of P30 IR *Hopx-Ascl1* CKOs are reduced in size compared to those of control IR mice.

## DISCUSSION

**Fig. 8.**
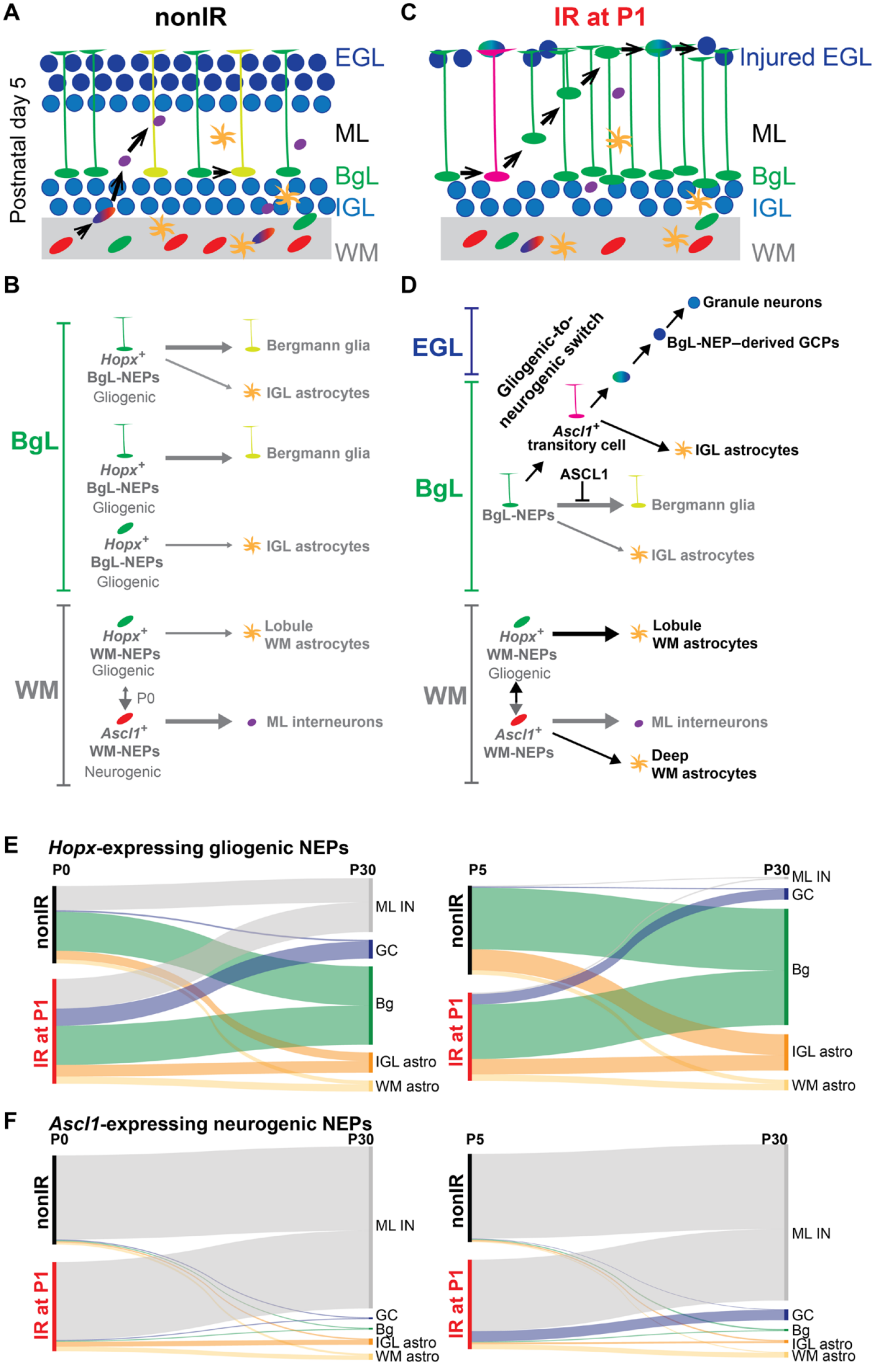
Schematic summary of the proposed NEP populations and lineages during homeostasis and repair. (**A** and **B**) On the basis of our results and published clonal analyses (*21*, *24*), we propose two major molecular NEP subtypes in the neonatal mouse cerebellum. At P5, one major molecular NEP subtype is astrogliogenic (*Hopx*-expressing) but divided into three subgroups in the BgL and one in the WM based on their position and whether they are lineage-restricted generating Bg or astrocytes, or are bipotent, generating both. The other major subtype is neurogenic (*Ascl1*-expressing) and resides only in the WM and generates INs. There are additional IN progenitor subgroups distinguished on the basis of their differentiation status (omitted for simplicity). Very rare oligodendrocytes that were detected in GIFM analyses were also omitted. Last, at birth, a WM-NEP population is bipotent generating INs and astrocytes (double vertical arrow). (**C** and **D**) Upon injury (black arrows), a subset of *Hopx*-derived BgL-NEPs turn on a neurogenic transcriptional program, which suppresses their normal Bg fate and allows them to undergo adaptive reprograming to generate new GCPs. Upon injury, there is also an increase in astrocyte production from *Ascl1*-expressing NEPs. For simplicity, the two lineage-restricted populations in the BgL are omitted. (**E** and **F**) Sankey plots summarizing the proportions of the cell types generated from the *Hopx*^+^ gliogenic (E) and *Ascl1*^+^ neurogenic NEPs (F) at P0 (left) and P5 (right) during homeostasis and upon injury. These plots do not distinguish the population of NEPs at P0 that coexpress *Hopx1* and *Ascl1* and the injury-induced gliogenic to neurogenic transitory cell state at P5 that is *Ascl1*^+^ in the BgL and shown in (D). GC, granule cell.

The neonatal mouse cerebellum has remarkable regenerative potential and cellular plasticity upon injury. Using scRNA-seq and GIFM, we defined two major NEP subtypes and the transcriptional signatures of their seven subpopulations. We also identified a key new transitory cellular state that is necessary for adaptive reprograming of BgL-NEPs into GCPs following EGL injury. Our results reveal that lineage propensity of NEPs (astroglial versus neural) is the primary molecular factor that differentiates NEP subtypes. At steady state, *Hopx*-expressing NEPs are proliferative, primarily astrogliogenic, and found in both the BgL and the WM, whereas the *Ascl1*-expressing NEPs are proliferative but restricted to the lobule WM and dedicated to making ML INs at P5. At P0, the two WM-NEP populations showed increased plasticity and likely contain a bipotent progenitor marked by *Hopx*. We found that, upon depletion of the GCPs, *Hopx*-expressing BgL-NEPs transition to a new state where proneural ventricular zone–bHLH genes are activated before their migration to the EGL. Furthermore, upon injury, we not only confirmed a delay in production of INs by *Ascl1*^+^ neurogenic NEPs using GIFM but also uncovered a subset switch to producing astrocytes, including some that become ectopically located in the deep WM. Last, given that ASCL1 is a pioneer transcription factor (*32*) and we found that it is required for full regeneration of the GCPs, we propose that ASCL1 is involved in erasing the astrogliogenic differentiation program of the *Hopx*^+^ BgL-NEPs, thus allowing them to acquire a GCP identity (Fig. 8).

An important question is whether within the astrogliogenic-NEPs, there are subpopulations dedicated to making only Bg or astrocytes, and if so, whether the latter are located in both the WM and the BgL. On the basis of our scRNA-seq analysis, there are two astrogliogenic-NEP subpopulations (N1 and N7) with one more related to astrocytes (N7) and the other to Bg (N1). Whereas Bg only are located in the BgL, *Hopx*-derived astrocytes are located in the IGL and lobule WM, which raises the question of whether *Hopx*^+^ BgL-NEPs and/or WM-NEPs generate astrocytes. For example, *Hopx*^+^ BgL-NEPs might generate IGL astrocytes and *Hopx*^+^ WM-NEPs generate the WM astrocytes. Elegant clonal labeling of apparently only BgL-NEPs using *Glast^CreER/+^; R26^Confetti/+^* mice and application of Tm to the surface of the P6 cerebellum in vivo found that the majority of clones had both Bg and astrocytes located in the IGL (~55%), although a large proportion was dedicated to making only Bg (~39%) and the rest to making only IGL astrocytes (*24*). This result indicates that WM astrocytes must be generated from WM-NEPs, likely the *Hopx*-expressing WM-NEPs that we identified. When WM progenitors were fluorescently labeled directly by injection of virus, some P3 to P5 WM-NEPs gave rise to WM astrocytes (*21*). Furthermore, our comparison of labeling before irradiation in IR and nonIR *Hopx-TdT* animals revealed preferential labeling of the BgL-NEPs that become *Ascl1*^+^ after injury, providing evidence that not all BgL-NEPs are equivalent in their extent of plasticity. Thus, molecular and lineage analyses show that WM astrocytes and Bg have distinct progenitors, whereas IGL astrocytes might come from both a bipotent progenitor and lineage-restricted progenitor in the BgL (summarized in Fig. 8).

At steady state, *Ascl1*^+^ cells are exclusively in the lobule WM and give rise almost exclusively to ML INs at P5, in agreement with previous findings at other postnatal ages (*25*, *31*). Unexpectedly, labeling at P0 in *Ascl1-TdT* animals resulted in an increase in astrocyte production in the lobules (IGL and WM) and the deep WM. We found that *Hopx-*expressing NEPs give rise to a very small number of INs at P5 and that the proportion and the number are higher when Tm is injected at P0, indicating that there could be a bipotent *Hopx*^+^ WM-NEP that produces INs in addition to astrocytes, likely through an *Ascl1*^+^ state in the WM. Further clonal analysis is required to resolve whether there are bipotent WM-NEPs that can give rise to both INs and WM astrocytes.

We showed that induction of the proneural ventricular zone–bHLH transcription factor ASCL1 in BgL-NEPs is a key step in their adaptive reprograming upon GCP death. Of importance, this *Ascl1*-driven gliogenic to neurogenic switch occurs without outside intervention such as the forced ectopic expression of proneural transcription factors required in other central nervous system regions (*34*, *35*, *37*). Further investigation is needed to identify the injury induced signal(s) that activate *Ascl1* expression in BgL-NEPs. Because up-regulation of *Ascl1* peaks 3 to 4 days after injury, the critical events required to produce the *Ascl1*^+^ transitory cell state must occur before P5. When *Ascl1-TdT* animals were labeled at P0, we did not observe labeled Bg-like cells or granule cells upon injury. These results confirm that the *Ascl1*^+^ BgL-NEP is an injury-induced cellular state. In another study, we showed that, upon depletion of Purkinje cells in the newborn cerebellum, although new Purkinje cells are generated, BgL-NEPs do not undergo adaptive reprogramming to produce them (*14*). Thus, the type of injury or cell type killed is instrumental in determining the downstream regenerative response of NEPs.

We previously showed that at the same time that the BgL-NEPs are generating new GCPs after irradiation, the lobule WM-NEPs reduce their proliferation and production of INs and, to a lesser extent, astrocytes (*12*). Our finding that expression of the WM-NEP marker *Igfbp5* is reduced after injury provides molecular evidence for such a response to EGL injury. An unexpected observation was that, after injury, *Ascl1*-expressing NEPs produce a notable number of astrocytes that populate the IGL and lobule WM, and some also migrate ectopically to the deep WM. Although the transitory *Ascl1*^+^ BgL-NEPs might be the source of the IGL astrocytes, it seems more likely that *Ascl1*^+^ WM-NEPs in the lobules have sufficient plasticity to switch to producing astrocytes in the lobules and deep WM upon injury given that *Ascl1*^+^ WM-NEPs at P0 produce some astrocytes. The reason for this neural-to-astroglial fate switch after injury and whether the same NEPs retain their neurogenic potential (i.e., are bipotent) or whether a distinct subpopulation of the *Ascl1*^+^ WM-NEPs become astrogliogenic remain to be explored.

A question remaining is whether some *Hopx*^+^ WM-NEPs contribute to the regeneration of granule cell precursors after cerebellar injury. Nevertheless, three findings indicate that BgL-NEPs are the main cells that undergo adaptive reprograming upon injury: (i) Upon injury, there is a significant increase in the number of *Ascl1*-expressing NEPs in the BgL that have a Bg-like morphology at P5; (ii) there is a lack of granule cell labeling upon irradiation using GIFM in *Ascl1-TdT* animals given Tm a day before irradiation (P0); and (iii) live imaging of *Ascl1*-expressing and *Nes-Cfp*^+^ NEPs shows them migrating from the BgL to the external granule cell layer (figs. S11) (*12*). Generation of tools that can selectively label WM-NEPs will be required to determine whether WM-NEPs also contribute to the regeneration process.

In conclusion, our studies provide molecular and cellular insights into the NEP subtypes in the neonatal cerebellum and the endogenous permissive mechanisms that support an astroglial-to-neural switch crucial for enhancing repair after brain injury. We identify a new transitory BgL-NEP population and demonstrate that ASCL1, which normally drives cerebellar IN production (*25*), is required for adaptive reprograming of BgL-NEPs to produce excitatory granule cells after death of GCPs. Furthermore, the mechanisms and cellular states that we identified may well have broader implications for gliogenic progenitors in other regions of the brain, and their potential responses to injury.

## METHODS

### Animals

All the mouse experiments were performed according to protocols approved by the Memorial Sloan Kettering Cancer Center’s Institutional Animal Care and Use Committee (protocol no. 07-01-001). Animals were housed on a 12-hour light/dark cycle and given access to food and water ad libitum.

The following mouse lines were used: *Nes-Cfp* (*12*, *49*), *Hopx^CreERT2^* (*50*), *Ascl1^CreERT2^* (*25*, *51*), *Ascl1^fl/fl^* (*25*, *52*) *Rosa26^lox-STOP-loxTdTomato^* (ai14, stock no. 007909, The Jackson Laboratory) (*53*). Animals were maintained on an outbred Swiss Webster background. Both sexes were used for analyses, and experimenters were blinded for genotypes whenever possible.

Tm (200 μg/g, Sigma-Aldrich) was injected subcutaneously at P0 or at P5. EdU (50 μg/g) was injected intraperitoneally at P7. Analysis of *Ascl1-TdT* and *Hopx-TdT* P30 brains that were not injected with Tm showed no ectopic TdT^+^ cells (fig. S6, A and B).

### Irradiation

P1 pups were anesthetized by hypothermia, and a single dose of 4-gray gamma irradiation was provided using an X-RAD 225Cx (Precision X-Ray) microirradiator in the Small Animal Imaging Core Facility at Memorial Sloan Kettering Cancer Center. The region of the cerebellum was targeted using a collimator with a 5-mm diameter.

### Tissue preparation and histological analysis

For P5 and younger anesthetized animals, brains were dissected and drop-fixed in 4% paraformaldehyde (PFA) for 24 to 48 hours at 4°C. Animals older than P5 were systemically perfused with ice-cold phosphate-buffered saline (PBS) followed by 4% PFA, following anesthesia with a ketamine (100 mg/kg) and xylaxine (10 mg/kg) cocktail. After dissection, brains were fixed for an additional 24 to 48 hours in 4% PFA. Fixed brains were switched to 30% sucrose in PBS. Once they sunk, brains were embedded in optimal cutting temperature compound (OCT) (Tissue-Tek) for cryosectioning. Fourteen-micrometer-thick sections were obtained using a cryostat (Leica, CM3050S) and stored at −20°C.

Hematoxylin and eosin (H&E) staining was performed for cerebellar area (size) measurements and assessment of the cerebellar cytoarchitecture.

Immunofluorescent analysis was performed on cryosections. Slides were allowed to warm to room temperature (RT) and washed once with PBS. One-hour blocking was performed at RT using 5% bovine serum albumin (Sigma-Aldrich) in PBS-T (PBS with 0.1% Triton X-100). For ASCL1 immunofluorescent analysis, Mouse on Mouse Blocking reagent (Vector Labs) was applied for 2 hours at RT to reduce the background. Slides were incubated with primary antibodies diluted in blocking solution at 4°C overnight (table S10). Slides were then washed with PBS-T (3 × 5 min) and incubated with fluorophore-conjugated secondary antibodies (1:500 in blocking buffer; Invitrogen). Hoechst 33258 (Invitrogen) was used to label the nuclei, and the slides were mounted with Fluoro-Gel mounting media (Electron Microscopy Sciences). To detect EdU, a Click-it EdU assay with Sulfo-Cyanine5 azide (Lumiprobe Corporation, A3330) was used.

### RNA in situ hybridization

Specimen treatment and hybridization were performed as previously described (*54*). Templates for the probes were in vitro transcribed either from polymerase chain reaction (PCR)–amplified complementary DNAs (cDNAs) obtained from neonatal cerebellar extracts or synthesized template DNA (GeneScript). An *Ascl1* probe was generated from a plasmid as previously described (*55*). Sequences of the probes are shown in table S11.

### Slice cultures and live imaging

P7 cerebella were dissected in ice-cold 1× Hanks’ balanced salt solution (HBSS) (Gibco) and embedded in 4% low–melting point agarose. Thick sagittal slices (250 μm) were obtained using a vibratome (Leica) and immediately placed on Millicell (Millipore) tissue culture inserts over Neurobasal media supplemented with 1× B27 and 1× N2 supplements (Gibco) and 2 mM l-glutamine and allowed to equilibrate for 30 min at 37°C in 5% CO_2_ before imaging. Image stacks were obtained every 3 to 4 min for 6 to 8 hours using LSM880 (Leica) with an environmental chamber (37°C with 5% CO_2_). Movies were processed using ZEN (Leica) and ImageJ [National Institutes of Health (NIH)] software (*12*, *56*).

### Image acquisition and analysis

Images were collected with a DM6000 Leica microscope or a Zeiss LSM 880 confocal microscope. Images were processed using ImageJ software (NIH).

Three near-midline sections for each animal were quantified and averaged for all the analyses shown. Quantification of the number of cells was performed on lobules 3 and 4/5 at P7 and on lobule 3 at P30. Boundaries of the lobules to be quantified were decided on the basis of straight lines drawn from the bases of the fissures. Cell densities were calculated by dividing the number of cells by the area of the lobule(s) quantified. Cerebellum area was measured on H&E-stained slides from three near-midline sections, and the values were averaged. The numbers of animals that were used in each quantification are denoted in the figure legends and where summary statistics are presented.

#### Quantification criteria used for fate mapping analyses

##### Neonatal analysis (2 days after Tm)

NEP populations are defined on the basis of their location in the WM + IGL, BgL + ML, or EGL. For the cells in the BgL, we further subdivided them based on their morphology: Cells with a Bg-like morphology (having processes reaching toward the pial surface) are classified as Bg-like NEPs and those cells without a process and morphology of migrating cells are classified as ML IN progenitors.

##### Adult analysis (P30)

Classification of the mature cell types for quantification was determined using a combination of cell-type specific markers, location, and morphology. The details for each TdT^+^ cell type are as follows:

*Granule cells:* NeuN^+^ cells in the IGL with small nuclei.

*ML INs:* PVALB^+^ cells in the ML.

*Bg:* S100β^+^ cells in the BgL with processes projecting to the pial surface (morphology).

*IGL and WM astrocytes:* S100B^+^ cells with a protoplasmic morphology.

*WM oligodendrocytes:* TdT^+^ cells with a distinct morphology, having fewer dendrites than astrocytes that are longer and symmetrically oriented and parallel to axonal tracts.

### Single-cell sequencing and data analysis

#### Sample preparation

Four male P5 nonIR and IR cerebella were dissected into ice-cold 1× HBSS (Gibco) and were pooled for downstream analysis. Two replicate experiments were performed. All the steps were performed on ice when possible. Cerebella were minced with a clean blade and then dissociated in Accutase (Innovative Cell Technologies) at 37°C for 10 to 15 min. Following dissociation, Accutase was washed out using neural stem cell media (Neurobasal, supplemented with N2, B27, and nonessential amino acids, Gibco). Following filtering through a cell strainer and trituration in media to single cells, cells were layered over a 5-ml density gradient (albumin-ovomucoid inhibitor solution, Worthington) and centrifuged at 70*g* for 6 min to remove debris. The cells were briefly treated with 1:2 1× red blood cell lysis buffer (Sigma-Aldrich) for 2 min at RT. Cells were washed twice (500*g*, 5 min at 4°C) and resuspended in 1× tris-buffered saline. Cells were stained with calcein acetoxymethyl live stain dye (1:500; Thermo Fisher Scientific) on ice for 30 min and were passed through a 40-μm cell strainer to remove any cell clumps before loading onto a microwell device.

#### Single-cell library preparation and sequencing

On-chip reverse transcription, cDNA amplification, and sequencing library preparation were performed as described previously (*39*, *45*, *57*). Briefly, reverse-transcription reactions were performed on DropSeq beads [Chemgenes, MACOSKO-2011-10 (V+)]. The microwell devices were scanned during the RNA capture to check for lysis efficiency. Our microwell method (*39*) does not lyse the nucleus as a means to reduce DNA contamination compared to other methods; therefore, analyses such as velocity that require unspliced pre-mRNA are not compatible with our dataset. Following PCR amplification, the libraries were prepared using a Nextera XT kit. DNA purifications were performed using AMPure XP beads. cDNA and library amount and quality were assessed using Qubit and a Bioanalyzer (Agilent). High-quality samples were sequenced on a NextSeq 500 using a High Output 75 cycle kit (read 1, 21 cycles; index, 8 cycles; read 2, 63 cycles).

#### Single-cell RNA sequencing data analysis

The sequencing reads were demultiplexed, aligned, and quantified as described previously (*57*). Twelve-nucleotide cell barcodes and eight- nucleotide unique molecular identifiers were extracted from read 1, and trimmed read 2 reads were aligned to a mouse genome (GRCm38, Gencode annotation vM10) using STAR v.2.5 (*58*). *Cfp* sequence was added in the annotation and denoted as NestinCFP in the expression matrices.

Unsupervised clustering was carried out as described previously (*45*, *59*). The clustering was performed on protein-coding genes only, including NESTINCFP. After computing cell-by-cell Spearman correlation matrix, a *k*–nearest neighbors graph was constructed with *k* set to 30. The resulting graph was used as input for Louvain clustering with Phenograph (*40*). *Fos*, a stress response gene associated with tissue dissociation, was removed from the cell clustering marker list (*60*). Cluster-specific genes were identified using a binomial-specificity test (*61*). UMAP was used for all data visualization (*62*).

To address coexpression and mutual exclusivity of genes detected in more than 25 cells, we calculated the marginal detection probabilities for each gene pair as described previously (*57*). The top 50 genes that were coexpressed with *Ascl1* in nonIR and IR NEPs (clustering shown in Fig. 1G) were identified for downstream analysis. The coexpression values were plotted as a scatter plot to identify condition-specific coexpressed genes.

We performed differential expression analysis between clusters and between nonIR and IR data as follows: First, we subsampled each pair of clusters to the same number of cells. Next, we subsampled the counts for each pair of clusters to the same average number of counts per cell, keeping only transcripts annotated as protein coding. We normalized the resulting subsampled count matrices using the computeSumFactors function (*63*) in scran and then conducted differential expression analysis with the Mann-Whitney *U* test as implemented in the mannwhitneyu function in SciPy and corrected the resulting *P* values using the Benjamini-Hochberg method as implemented in the multipletests function in the Python module StatsModels. The resulting gene lists were preranked on the basis of on the effect size and were inputted to GSEA (*64*) using the following parameters: xtools.gsea.GseaPreranked -scoring_scheme classic –setmin 10 –setmax 1000 –nperm 1000.

Because velocity analysis could not be performed on our dataset, pseudotemporal ordering of the IR NEPs from clusters N1, N6, and N7 (559 cells) was performed using Monocle3 (*65*). Analysis was performed using the latent semantic indexing method, and branch length was set to 60.

### Statistical analysis

Prism (GraphPad) was used for all statistical analysis. Statistical tests performed in this study were Student’s two-tailed *t* test, two-way ANOVA, followed by post hoc analysis with Tukey’s multiple comparison tests. *P* values of the relevant post hoc analyses are shown in the figures and in table S12. The statistical significance cutoff was set at *P* < 0.05, and the data are presented as means ± SD of the mean. *F*-statistics and *P* values are stated in the figure legends, and relevant post hoc comparisons are shown in the figures. *n* ≥ 3 mice were used for each experiment, and the sample size for each experiment is stated in the figure legends and the text.

## Supplementary Materials

### This PDF file includes:

Figs. S1 to S13 Tables S3 to S5 and S9 to S12 Legends for movies S1 and S2 Legends for tables S1, S2 and S6 to S8

## Notes

View/request a protocol for this paper from *Bio-protocol*.
