# Supplementary material for: Injury-induced ASCL1 expression orchestrates a transitory cell state required for repair of the neonatal cerebellum
Source: Sci Adv. 2021 Dec 8;7(50):eabj1598. doi: 10.1126/sciadv.abj1598 (PMC8654303; doi:10.1126/sciadv.abj1598)
Supplement: Supplementary file 1 — Figs. S1 to S13 Tables S3 to S5 and S9 to S12 Legends for movies S1 and S2 Legends for tables S1, S2 and S6 to S8 [file sciadv.abj1598_sm.pdf]

Science Advances
AAAS

Supplementary Materials for

Injury-induced ASCL1 expression orchestrates a transitory cell state required for repair of the neonatal cerebellum

N. Sumru Bayin*, Dogukan Mizrak, Daniel N. Stephen, Zhimin Lao,
Peter A. Sims, Alexandra L. Joyner*

*Corresponding author. Email: joynera@mskcc.org (A.L.J.); bayinn@mskcc.org (N.S.B.)

Published 8 December 2021, *Sci. Adv.* **7**, eabj1598 (2020)
DOI: 10.1126/sciadv.abj1598

The PDF file includes:

Figs. S1 to S13
Tables S3 to S5 and S9 to S12
Legends for movies S1 and S2
Legends for tables S1, S2 and S6 to S8

Other Supplementary Material for this manuscript includes the following:

Movies S1 and S2
Tables S1, S2 and S6 to S8

Supplementary Figures and Figure Legends

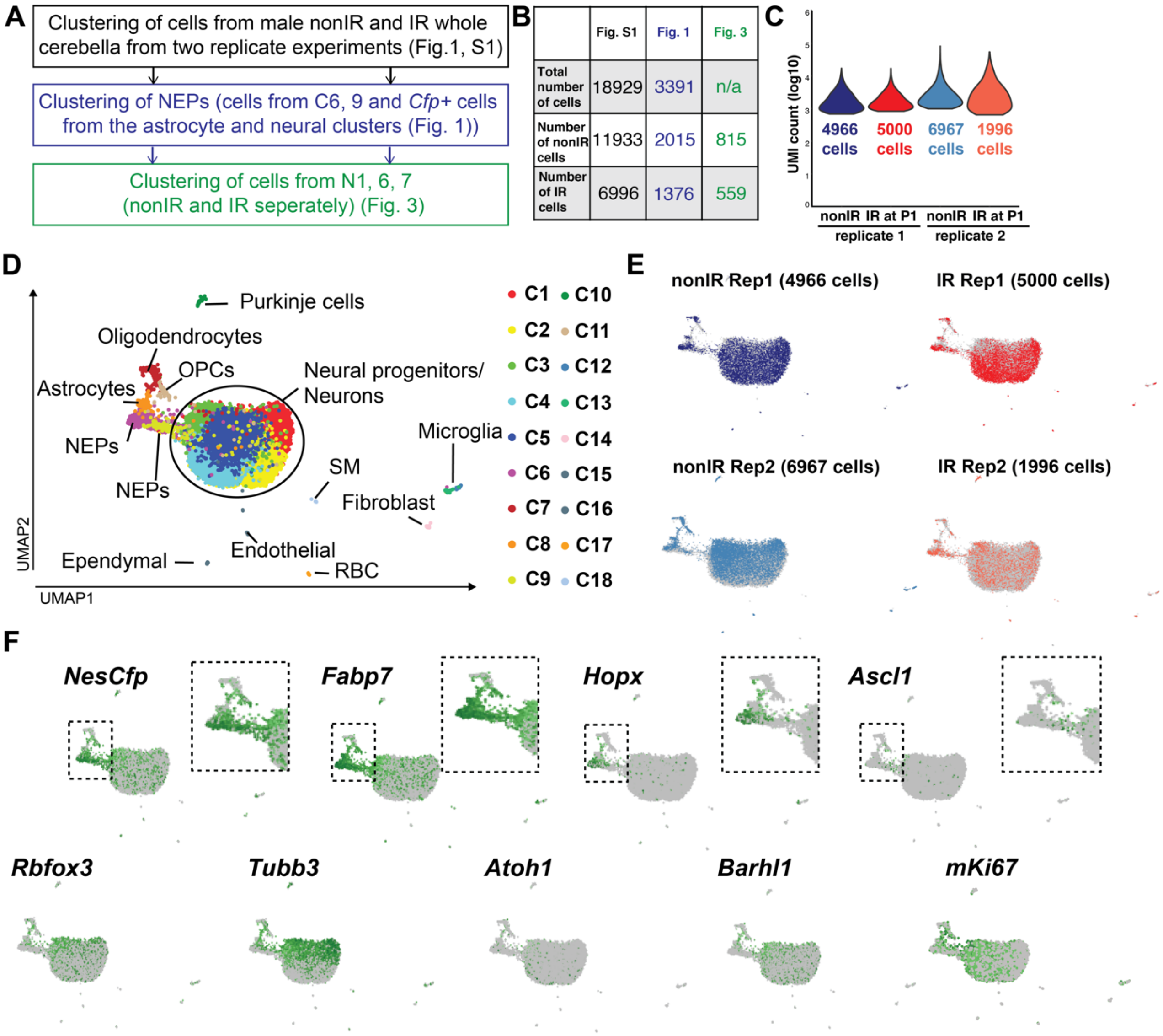

Fig. S1. Summary of the scRNA-seq experiments and the initial clustering of the 18,929 cells from nonIR and IR cerebella at P5. A. Schematic explaining the clustering logic used for the scRNA-seq data analysis. **B.** Table summarizing the number of cells from nonIR and IR that were used for each analysis and the figures where the analyses are represented. **C.** Distribution of the UMI counts ($\log_{10}$) of the samples sequenced that passed quality control. **D-E.** UMAP visualization of the clustering of 18,929 cells shown by cell types (clusters, D) and by condition across two replicates (E) 18 clusters were

detected using Phenograph. *Nes-Cfp* was enriched in clusters 3, 6-11 (Table S1), insets shown the *Cfp* enriched clusters. **F.** Expression levels of *Nes-Cfp* and other gliogenic and neurogenic lineage makers and mKi67 projected on UMAP (scale shows the log transformed CPM (counts per million) where the darker colors show higher expression). SM: smooth muscle, RBC: red blood cell, OPC: oligodendrocyte progenitor cells

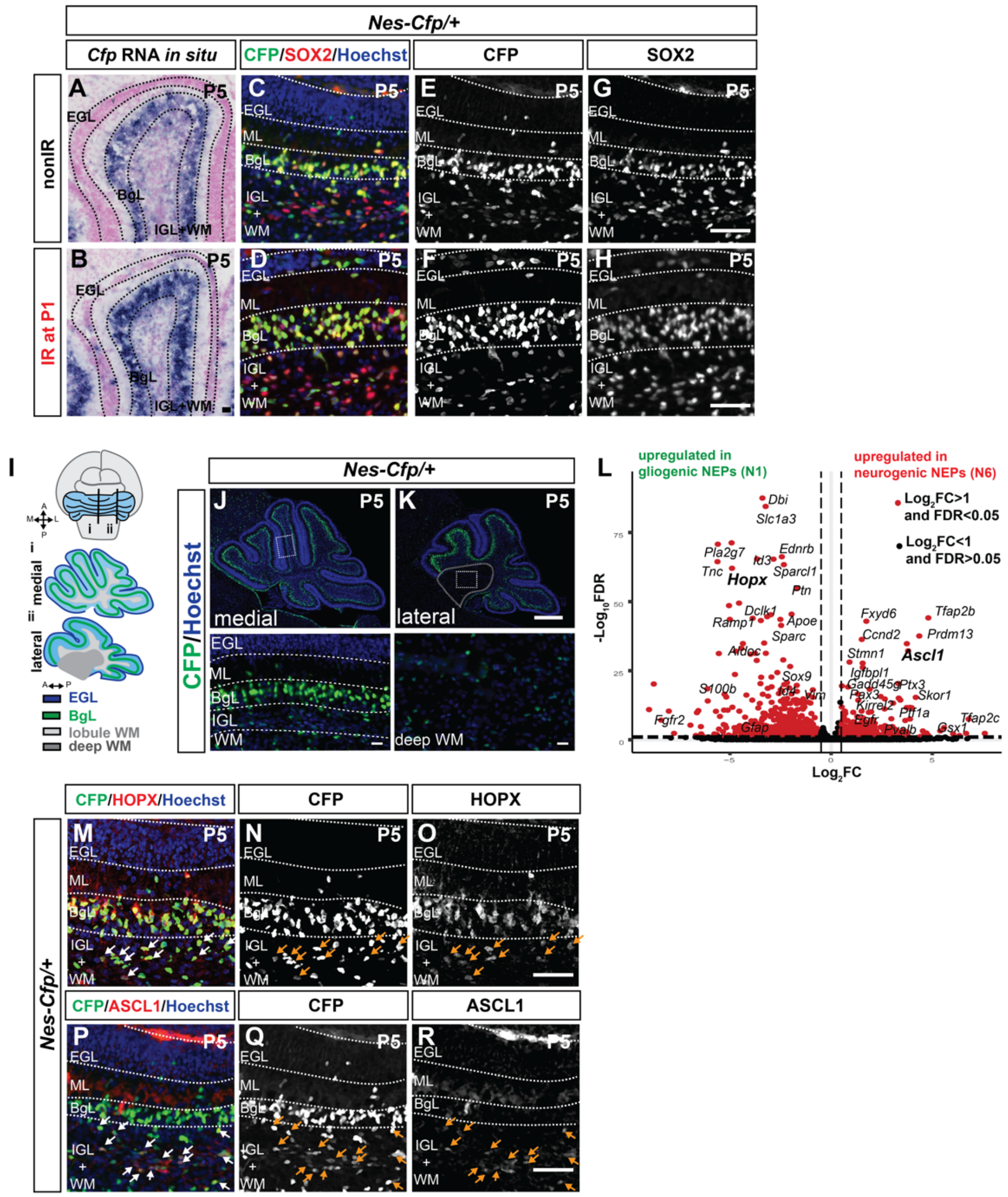

Fig. S2. Validation of the *Nes-Cfp* reporter at P5 on nonIR and IR cerebellar sections and characterization of the expression patterns of the gliogenic (HOPX) and neurogenic (ASCL1) NEP markers. A-B. RNA in situ hybridization for *Cfp* on sections from nonIR and IR P5 *Nes-Cfp/+* cerebella. **C-H.** IF analysis of CFP and SOX2 on sections from nonIR (C, E, G) and IR (D, F, H) P5 *Nes-Cfp/+* cerebella shows high overlap for CFP and SOX2 (pan-NEP/astroglial marker). **I.** Schematics showing the sagittal levels

of the medial and lateral cerebellum used for analysis. **J-K.** IF analysis of P5 *Nes-Cfp*+ nonIR cerebella at medial (J) and lateral (K) levels showing the distinct localization of NEPs within the cerebellum. **L.** Volcano plots showing the differentially expressed genes (FC: fold change and FDR: false discovery rate, Table S2) obtained from the comparison of the undifferentiated gliogenic (N1) and the neurogenic (N6) NEPs. **M-R.** IF analysis of HOPX (M-O) and ASCL1 (P-R) on sections from P5 *Nes-Cfp/*+ cerebella show that all HOPX+ and ASCL1+ cells are CFP positive (arrows show double positive cells in the IGL and WM). EGL: external granule layer, ML: molecular layer, BgL: Bergmann glia layer, IGL: internal granule layer, WM: white matter. Scale bars: 50μm, J-K: 500μm,

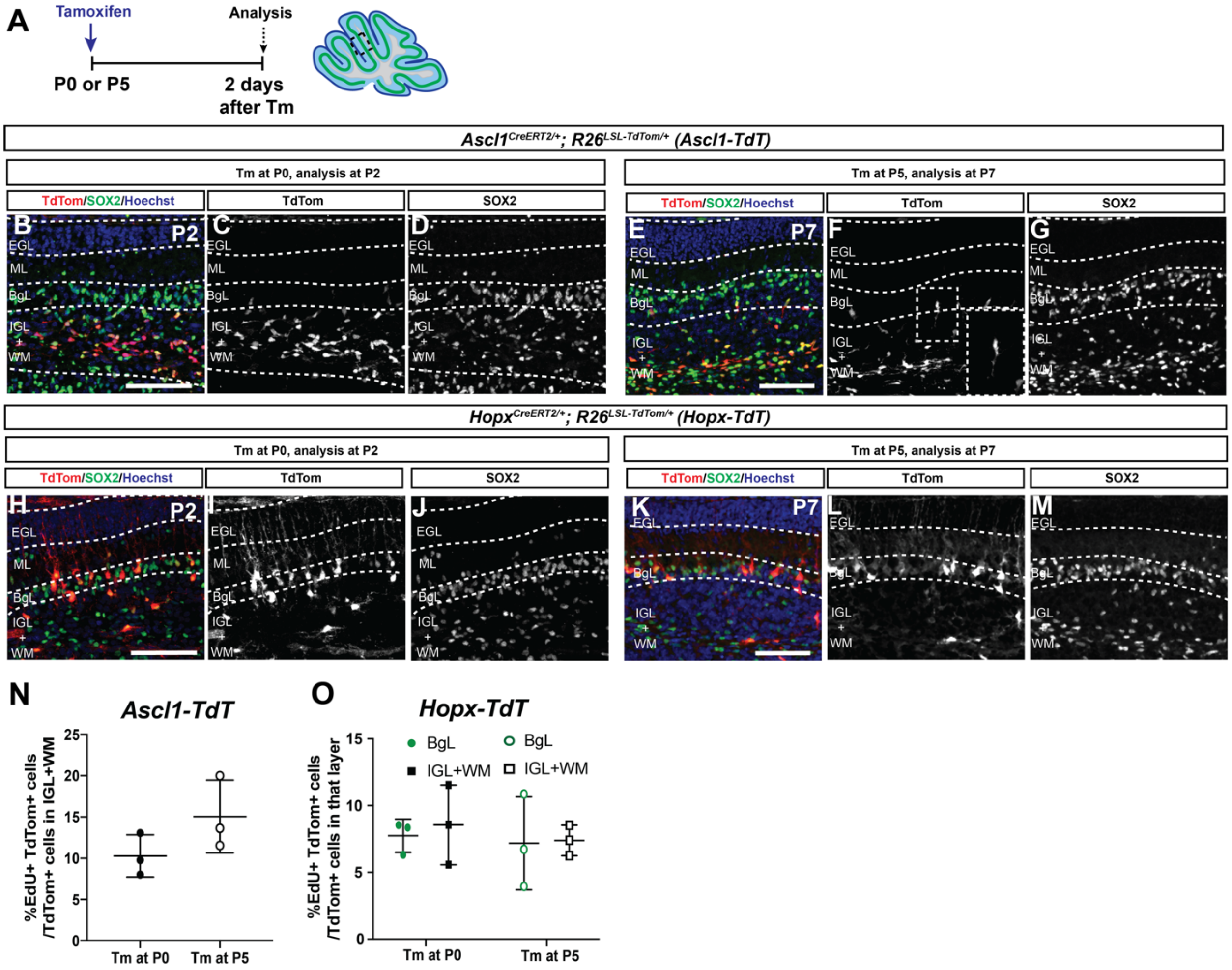

Fig. S3. Short term genetic inducible fate mapping identifies the localization of neurogenic and astrogliogenic NEP subtypes and NEP subtypes are proliferative. **A**. Experimental plan and the region of the cerebellum shown in the images. **B-G**. IF analysis used for the quantification of the TdT+ cells by layer 2 days after Tm injection (B-D: Tm at P0, E-G: Tm at P5) in *Ascl1-TdT* animals (n=3 brains/age). **H-M.** IF analysis used for the quantification of the TdT+ cells by layer 2 days after Tm injection (H-J: Tm at P0, K-M: Tm at P5) in *Hopx-TdT* animals (n=3 brains/age). SOX2 is used to identify all NEPs. N-O**.** Proliferation of the NEPs is analyzed by injecting EdU 1 hour before sacrifice. Quantification of the percentage of EdU+ TdT+ cells in all TdT+ cells in the respective

layers showed that both *Ascl1-TdT+* (N) and *Hopx-TdT+* (O) cells are proliferative at P0 and at P5, confirming their progenitor status (n=3 brains/condition). Scale bars: 100 μm, EGL: external granule layer, ML: molecular layer, BgL: Bergmann glia layer, IGL: internal granule layer, WM: white matter

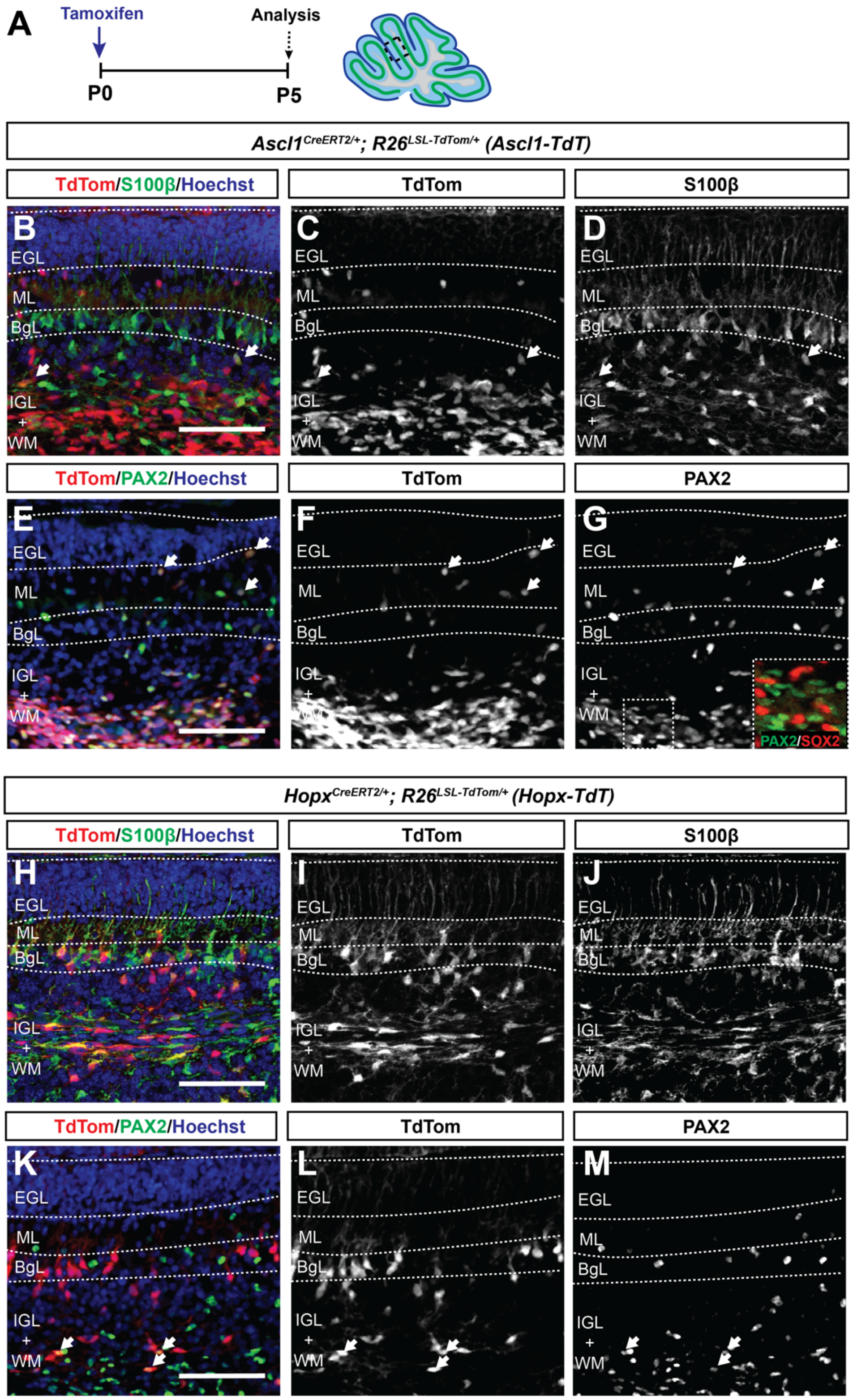

Fig. S4. Analysis of the progeny of *Ascl1-TdT*+ and *Hopx-TdT*+ cells that were given Tm at P0 and analyzed at P5 for lineage markers shows the lineage propensities of

the NEP subtypes. A. Experimental plan and the region of the cerebellum shown in the images. **B-G**. IF analysis of *Ascl1-TdT* cerebellar that were given Tm at P0 and analyzed at P5 shows that majority of the TdT+ cells are in the WM and IGL and are PAX2+ (immature interneuron marker, E-G), whereas rare cells showed co-expression with S100β (glial marker, arrows B-D). Some of the TdT+ cells that are migrating (in the BgL or ML) showed costaining with PAX2 (arrows, (E-G)). **H-M.** IF analysis of *Hopx-TdT* cerebellar that were given Tm at P0 and analyzed at P5 shows that majority of the TdT+ cells are in the BgL, some are in the WM and IGL and most of the TdT+ cells are S100β+ (H-J), whereas rare cells showed co-expression with PAX2 in the WM (arrows K-M). EGL: external granule layer, ML: molecular layer, BgL: Bergmann glia layer, IGL: internal granule layer, WM: white matter. Scale bars: 100μm

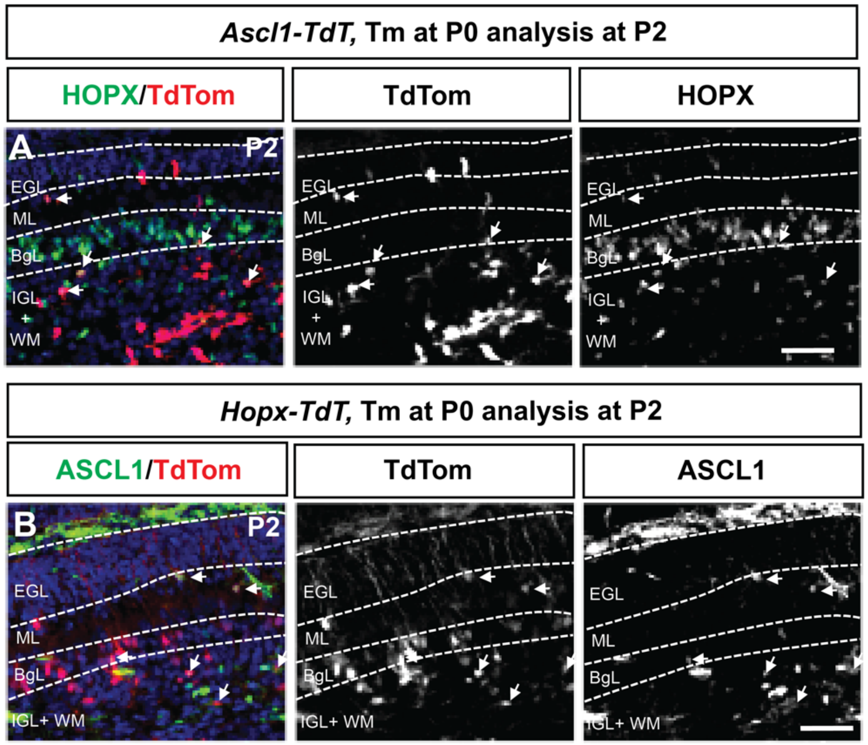

Fig. S5. Overlap between the *Ascl1*+ neurogenic and *Hopx*+ astrogliogenic NEPs at P0. A-B. Short term genetic inducible fate mapping and IF analysis for HOPX and ASCL1 in the *Ascl1-TdT* (A) and *Hopx-TdT* (B) cerebella, respectively, that were administered Tm at P0 and analyzed at P2 shows ~10% overlap between the HOPX+ and ASCL1+ NEPs, mostly in the IGL + WM and migrating interneurons progenitors in the ML (n=3 brains/genotype). Arrows show marker positive TdT+ cells. Scale bars: 50µm, EGL: external granule layer, ML: molecular layer, BgL: Bergmann glia layer, IGL: internal granule layer, WM: white matter

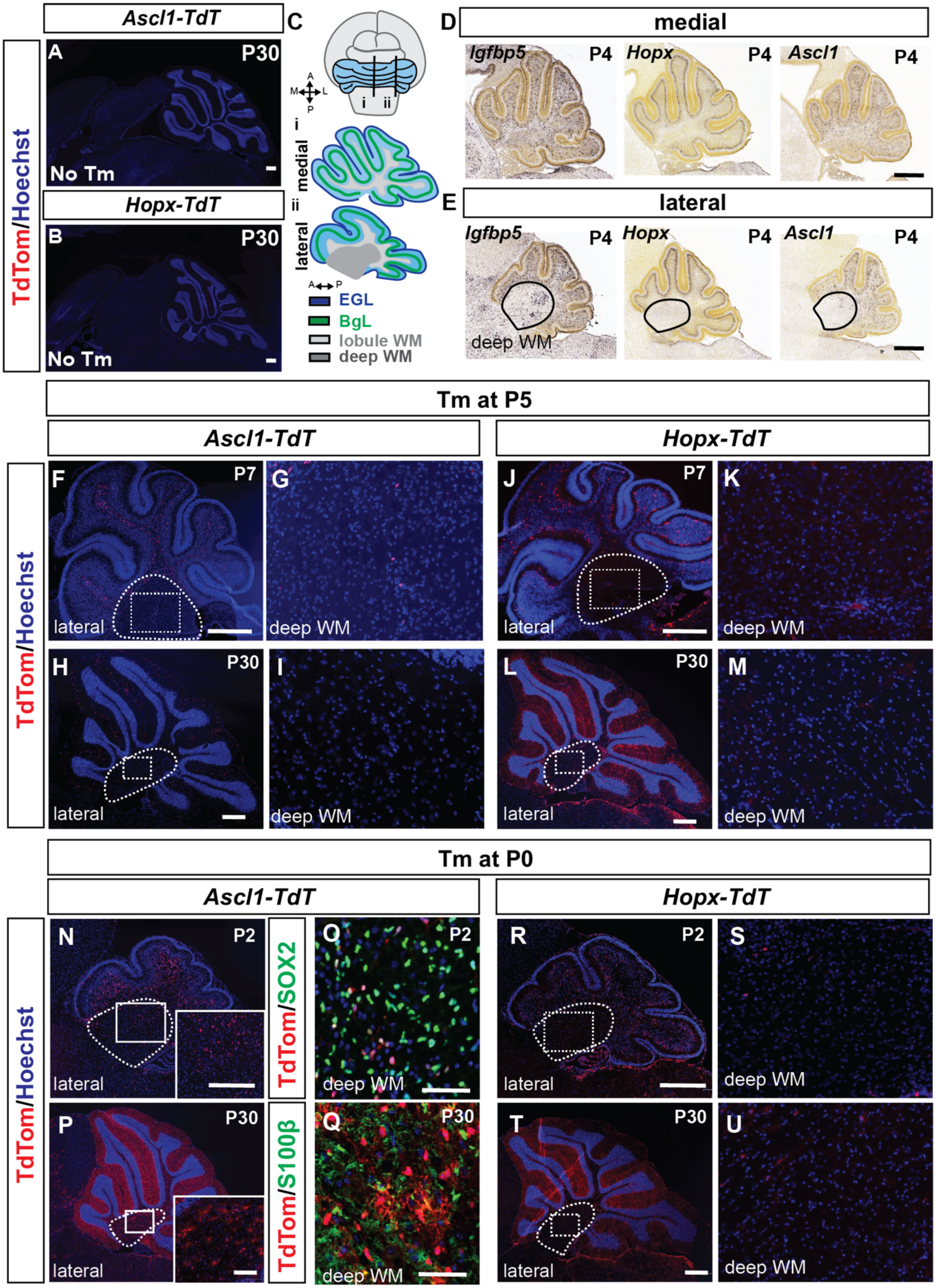

Ascl1-TdT
A
P30
No Tm
Hopx-TdT
B
P30
No Tm
TdTom/Hoechst
C
A
M
L
P
i
ii
i
medial
ii
lateral
A P
EGL
BgL
lobule WM
deep WM
D
medial
Igfbp5
P4
Hopx
P4
Ascl1
P4
E
lateral
Igfbp5
P4
Hopx
P4
Ascl1
P4
deep WM
Tm at P5
Ascl1-TdT
Hopx-TdT
TdTom/Hoechst
F
P7
lateral
G
deep WM
H
P30
lateral
I
deep WM
J
P7
lateral
K
deep WM
L
P30
lateral
M
deep WM
Tm at P0
Ascl1-TdT
Hopx-TdT
TdTom/Hoechst
N
P2
lateral
TdTom/SOX2
O
P2
deep WM
P
P30
lateral
TdTom/S100β
Q
P30
deep WM
R
P2
lateral
S
deep WM
T
P30
lateral
U
deep WM

Fig. S6. Genetic inducible fate mapping of *Hopx*+ and *Ascl1*+ NEPs and *RNA in situ* hybridization shows that the progeny of *Hopx*+ and *Ascl1*+ NEPs are restricted to lobules at P5, whereas *Ascl1*+ NEPs give rise to astrocytes in the deep WM at P0. A-B. Analysis of P30 *Ascl1-TdT* (A) and *Hopx-TdT* (B) mice that were not given Tm show no ectopic labeling due to recombination. **C.** Schematics showing the level of sagittal levels in the medial and lateral cerebellum used for analysis. **D-E.** Allen Brain Atlas P4 RNA *in situ* hybridization data showing the expression patterns of marker genes in clusters identified by scRNA-seq in the medial (D) and the lateral (E) cerebellum**. F-M**. Analysis of sections of the lateral cerebellum and deep WM of *Ascl1-TdT* and *Hopx-TdT* mice that were given Tm at P5 and analyzed at P7 (F-G, J-K) and at P30 (H-I, L-M) shows no TdT+ cells in the deep WM. **N-U**. Analysis of sections of the lateral cerebellum and deep WM of *Ascl1-TdT* and *Hopx-TdT* mice that were given Tm at P0 and analyzed at P2 (N-O, R-S) and at P30 (P-Q, T-U) shows TdT+ cells in the deep WM only in the *Ascl1-TdT*+ cerebella. *Ascl1-TdT*+ cells are SOX2+ at P2 (O) and show mostly astroglial morphology and are S100β+ at P30 (Q). Scale bars: 500μm, O, Q: 50μm

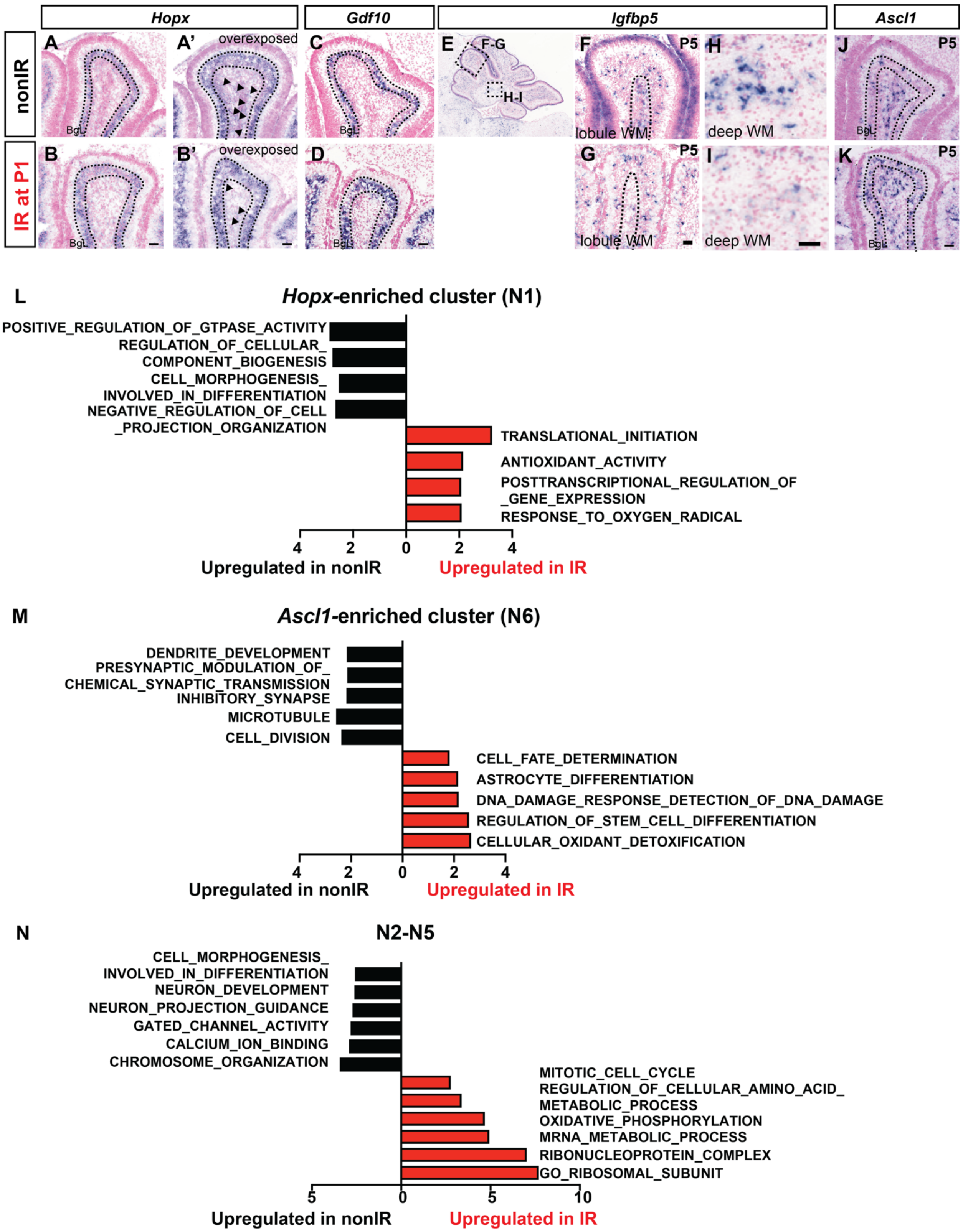
Hopx
Gdf10
Igfbp5
Ascl1
nonIR
IR at P1
A
A'
overexposed
C
E
F-G
H-I
F
P5
H
J
P5
BgL
lobule WM
deep WM
B
B'
overexposed
D
G
P5
I
K
P5
L
Hopx-enriched cluster (N1)
POSITIVE_REGULATION_OF_GTPASE_ACTIVITY
REGULATION_OF_CELLULAR_
COMPONENT_BIOGENESIS
CELL_MORPHOGENESIS_
INVOLVED_IN_DIFFERENTIATION
NEGATIVE_REGULATION_OF_CELL
_PROJECTION_ORGANIZATION
TRANSLATIONAL_INITIATION
ANTIOXIDANT_ACTIVITY
POSTTRANSCRIPTIONAL_REGULATION_OF
_GENE_EXPRESSION
RESPONSE_TO_OXYGEN_RADICAL
4 2 0 2 4
Upregulated in nonIR
Upregulated in IR
M
Ascl1-enriched cluster (N6)
DENDRITE_DEVELOPMENT
PRESYNAPTIC_MODULATION_OF_
CHEMICAL_SYNAPTIC_TRANSMISSION
INHIBITORY_SYNAPSE
MICROTUBULE
CELL_DIVISION
CELL_FATE_DETERMINATION
ASTROCYTE_DIFFERENTIATION
DNA_DAMAGE_RESPONSE_DETECTION_OF_DNA_DAMAGE
REGULATION_OF_STEM_CELL_DIFFERENTIATION
CELLULAR_OXIDANT_DETOXIFICATION
4 2 0 2 4
Upregulated in nonIR
Upregulated in IR
N
N2-N5
CELL_MORPHOGENESIS_
INVOLVED_IN_DIFFERENTIATION
NEURON_DEVELOPMENT
NEURON_PROJECTION_GUIDANCE
GATED_CHANNEL_ACTIVITY
CALCIUM_ION_BINDING
CHROMOSOME_ORGANIZATION
MITOTIC_CELL_CYCLE
REGULATION_OF_CELLULAR_AMINO_ACID_
METABOLIC_PROCESS
OXIDATIVE_PHOSPHORYLATION
MRNA_METABOLIC_PROCESS
RIBONUCLEOPROTEIN_COMPLEX
GO_RIBOSOMAL_SUBUNIT
5 0 5 10
Upregulated in nonIR
Upregulated in IR

Fig. S7. RNA *in situ* analysis of P5 nonIR and IR cerebella for NEP markers and GSEA analysis on differentially expressed genes between nonIR and IR cells within NEP subtypes show the injury induced changes in NEP subtypes. A-K. RNA *in situ* analysis of P5 nonIR and IR brains for BgL-NEP (*Hopx, Gdf10,* A-D), WM-NEPs (*Igfbp5*, E-I) and neurogenic-NEP (*Ascl1,* J-K) markers**. A'-B'.** Overexposed *Hopx* RNA *in situ* analysis shows the *Hopx*-expressing cells in the WM (arrowheads). **L-N.** GSEA analysis (NES≥2, FDR<0.1) of differentially expressed genes between the nonIR and IR cells within the *Hopx*-enriched (N1, L), *Ascl1*-enriched (N6, M) clusters and clusters N2-5 (N) from Fig. 1. x-axis shows normalized enrichment score (NES). Scale bars: 50μm, BgL: Bergmann glia layer, WM: white matter

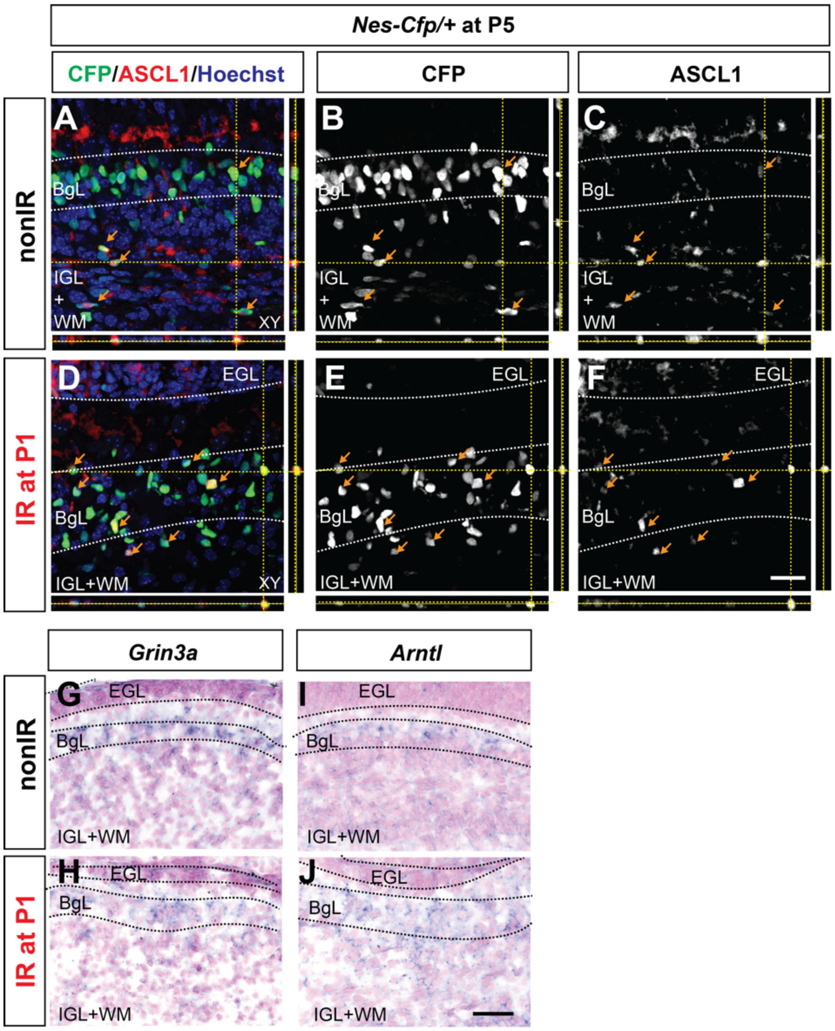

Fig. S8. ASCL1 is ectopically expressed in the BgL upon irradiation and genes that are co-expressed with *Ascl1* in the IR NEPs are expressed in the BgL. A-F. IF analysis of ASCL1 and CFP on cerebellar sections from P5 *Nes-Cfp/+* nonIR (A-C) or IR (D-E) cerebella. Orthogonal projections of confocal z-stacks are presented. Arrows show the ASCL1 and CFP double positive cells. **G-J.** RNA *in situ* hybridization for *Grin3a* (G-H) and *Arntl* (I-J) on cerebellar sections from P5 *Nes-Cfp/+* nonIR (G, I) or IR (H, J) cerebella. Scale bars: 50µm. EGL: external granule layer, ML: molecular layer, BgL: Bergmann glia layer, IGL: internal granule layer, WM: white matter

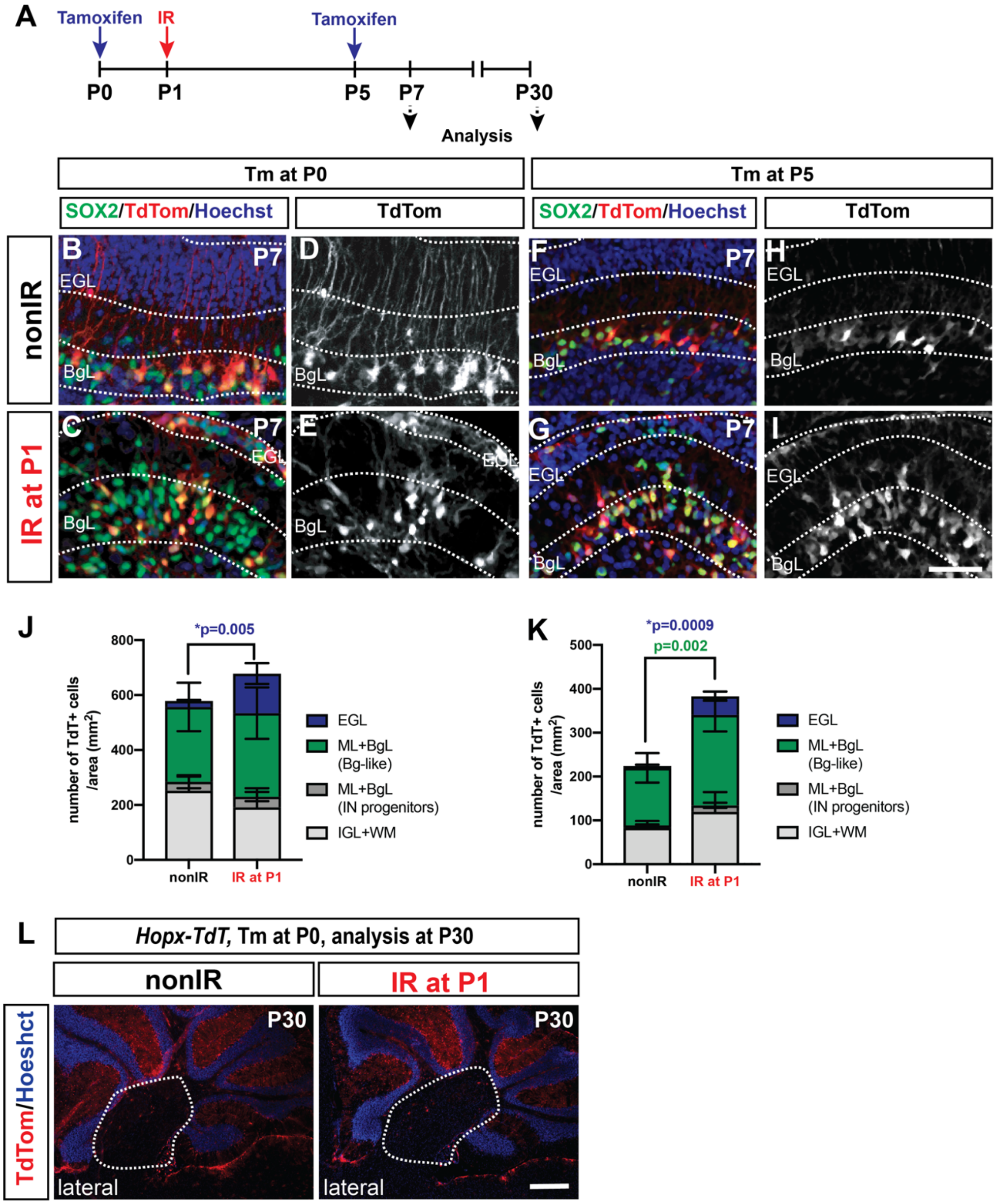

Fig. S9. ***Hopx*-expressing NEPs migrate to external granule layer upon injury and do not generate astrocytes in the deep WM after irradiation. A.** Experimental plan. **B-I.** IF analysis of P7 nonIR (B, D, F, H) and IR (C, E, G, I) *Hopx-TdT* brains that were given Tm at P0 (B-E) or at P5 (F-I). **J.** Quantification of the density of TdT+ cells in nonIR and

IR *Hopx-TdT* brains that were given Tm at P0, analyzed at P7 (n=3/condition, EGL: Student's t-test, p=0.005). **K.** Quantification of the density of TdT+ cells in nonIR and IR *Hopx-TdT* brains that were given Tm at P5, analyzed at P7 (n≥3/condition, Two-way ANOVA, $F_{(1, 20)}$=19.5, p=0.0003, *EGL: Student's t-test, p=0.0009) **L.** Analysis of *Hopx-TdT* P30 nonIR and IR mice at P1 shows no TdT+ cells in the deep WM of the lateral cerebellum. Scale bars: B-I: 50μm, J: 500μm

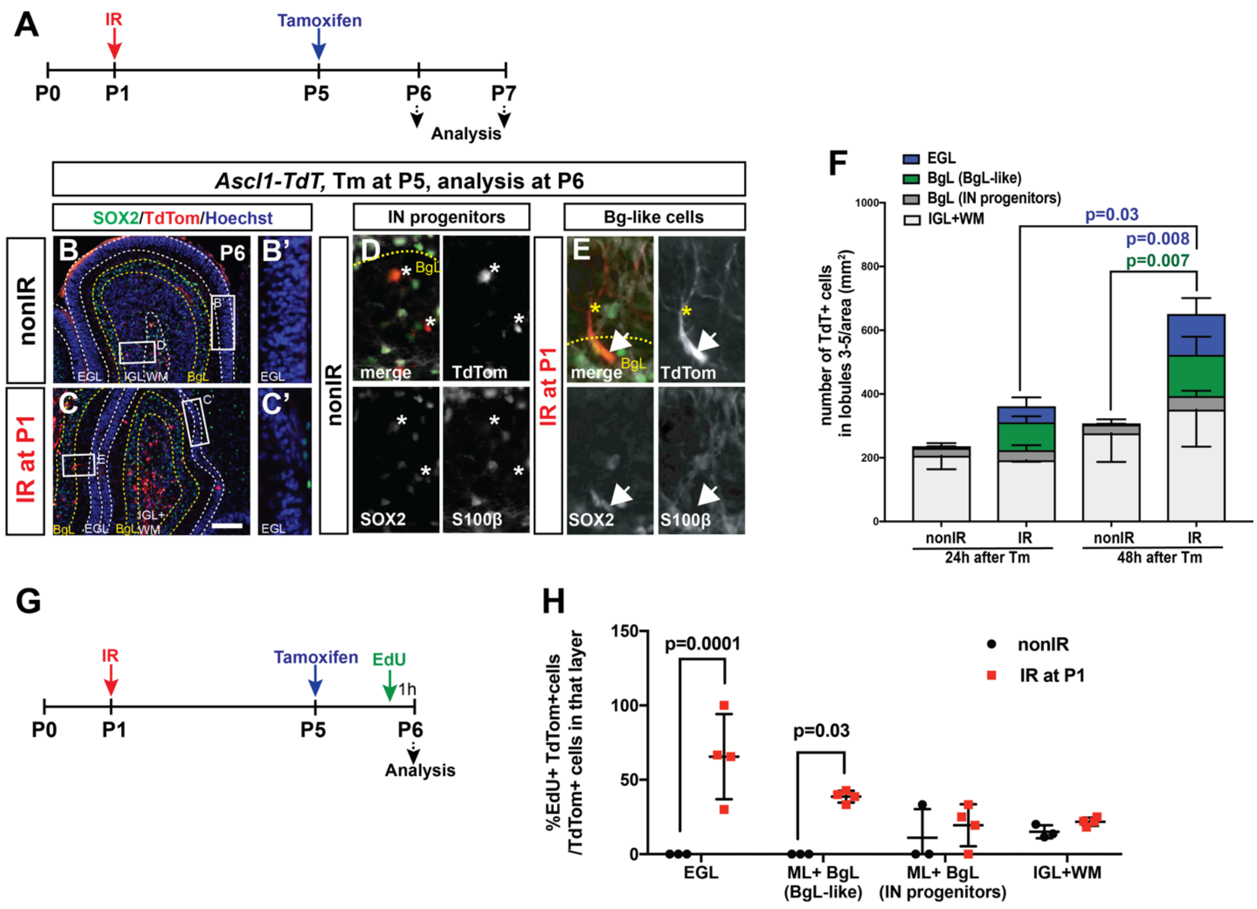

Fig. S10. A Proliferative transitory *Ascl1*+ BgL-NEP emerges upon injury. A, G. Experimental plan. **B-E.** IF analysis of *Ascl1-TdT* brains at P6 (24h after Tm at P5. Insets in B' and C' show the EGL and the insets in D and E show migrating interneuron progenitors (asterisks; D) and a SOX2+ S100β- BgL-like *Ascl1-TdT* cell (arrows; E), respectively. Yellow asterisks show the pial projection of the TdT+ cells. **F.** Quantification of the density of the TdT+ cells in the different layers of the cerebellum at P6 and P7 in nonIR and IR *Ascl1-TdT*+ mice given Tm at P5 (BgL-like cells were identified by their pial projections (n=4, Two-way ANOVA, all layers: $F_{(3,32)}$=12.89, p<0.0001, EGL: $F_{(1,8)}$=23.75, p=0.001). **H.** Quantification of the percentages of EdU+ TdT+ cells per all TdT+ cells in each layer in *Ascl1-TdT* animals at P6 in nonIR and IR P1 pups shows that the

neurogenic-NEPs at steady state and the TdT+ BgL-NEPs and the TdT+ cells in the external granule layer cells are proliferative upon irradiation (n=3 for nonIR and n=4 for IR, Two-way ANOVA, $F_{(3,20)}=31.28$, $p<0.0001$). Scale bars: 100µm, EGL: external granule layer, ML: molecular layer, BgL: Bergmann glia layer, IGL: internal granule layer, WM: white matter

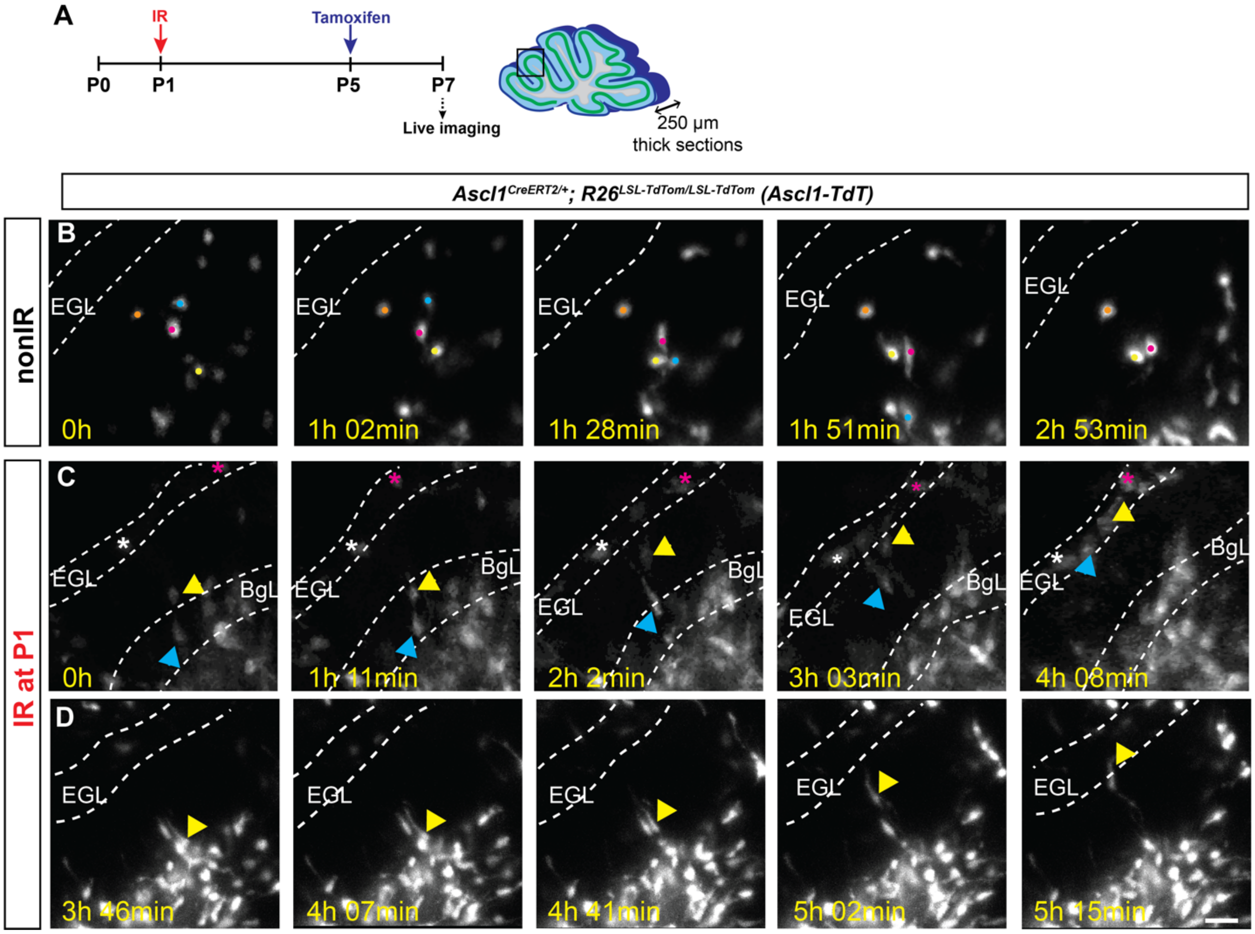

Fig. S11. Time lapse images showing *Ascl1-TdT*+ BgL-NEPs migrating to external granule layer after IR. A. Schematic of the experimental plan. **B-D**. Examples of time-lapse live images of TdT+ cells from cerebellar sections obtained from P7 nonIR (B) or IR (C-D) *Ascl1-TdT* pups. Scale bars: 25µm, EGL: external granule layer

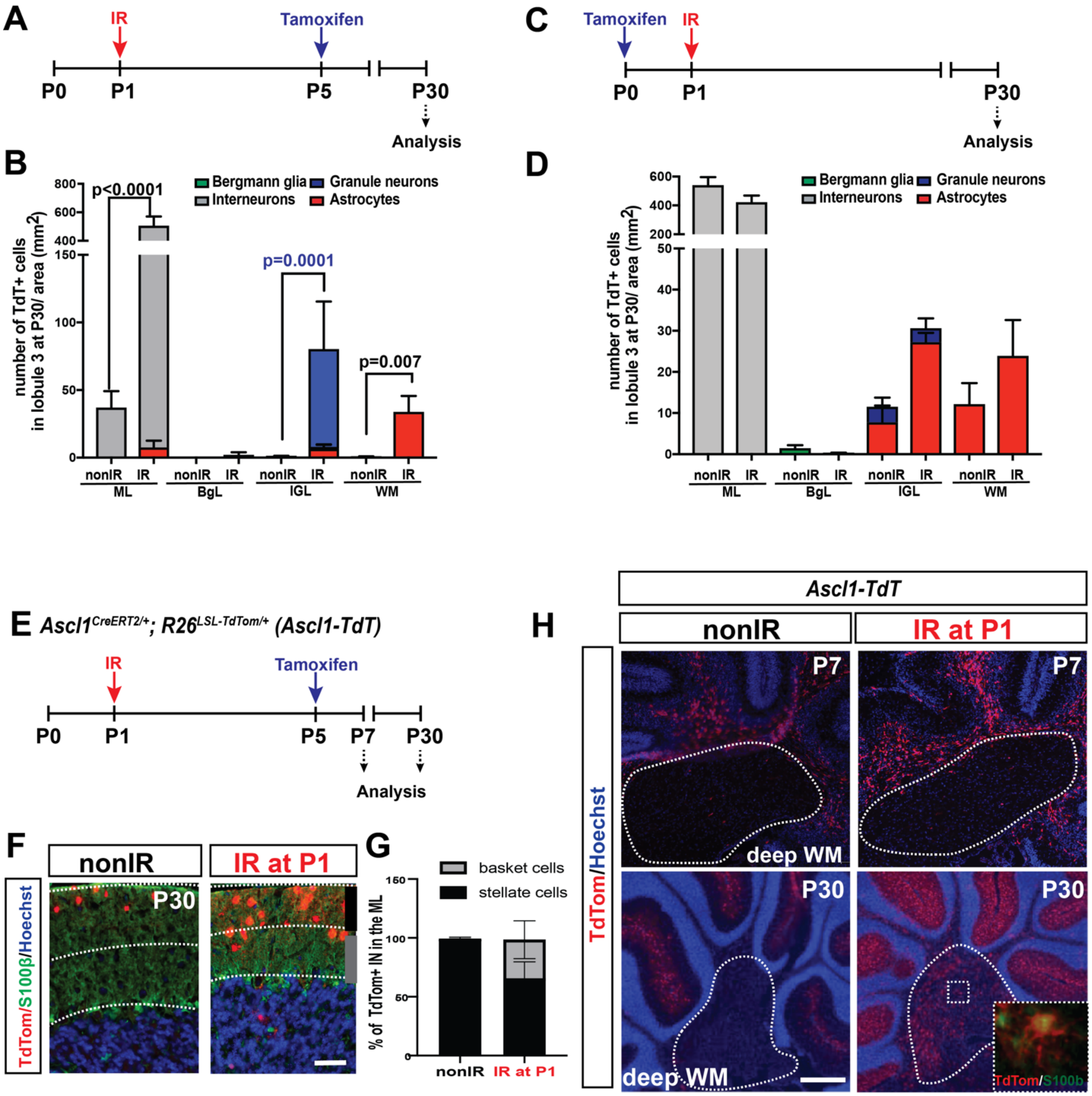

Fig. S12. Ascl1-TdT+ cells give rise to granule cells, ML interneurons and WM astrocytes when labelled after the injury but not before. A, C, E. Schematics explaining the timelines of the experiments. **B, D.** Quantification of the density of the TdT+ cells by cell type when Tm is administered at P5 (B) or at P0 (D) in the different layers of the cerebellum of P30 nonIR and IR *Ascl1-TdT* animals (B: n=3/condition, Two-way ANOVA, $F_{(7,14)}$=137.9, p<0.0001, WM astrocytes: Student's t-test, p<0.007, D:

n≥3/condition, Two-way ANOVA, $F_{(7,83)}=275.4$, $p<0.0001$). **E.** Experimental plan. **F-G.** IF analysis of the TdT+ cells in the ML of the P30 nonIR and IR *Ascl1-TdT* animals (F) and the quantification of the proportions of Basket neurons (early born, lower ML) and Stellate neurons (later born, upper ML) shows a delay in the production of ML interneurons in the mice irradiated at P1. **H.** IF analysis of the lateral cerebellum of nonIR and IR at P1 *Ascl1-TdT* animals shows TdT+ astrocytes in the deep WM (S100β+) at P30 but not at P6, suggesting the cells migrate from the lobule WM. Scale bars: 500µm, except for F: 50µm

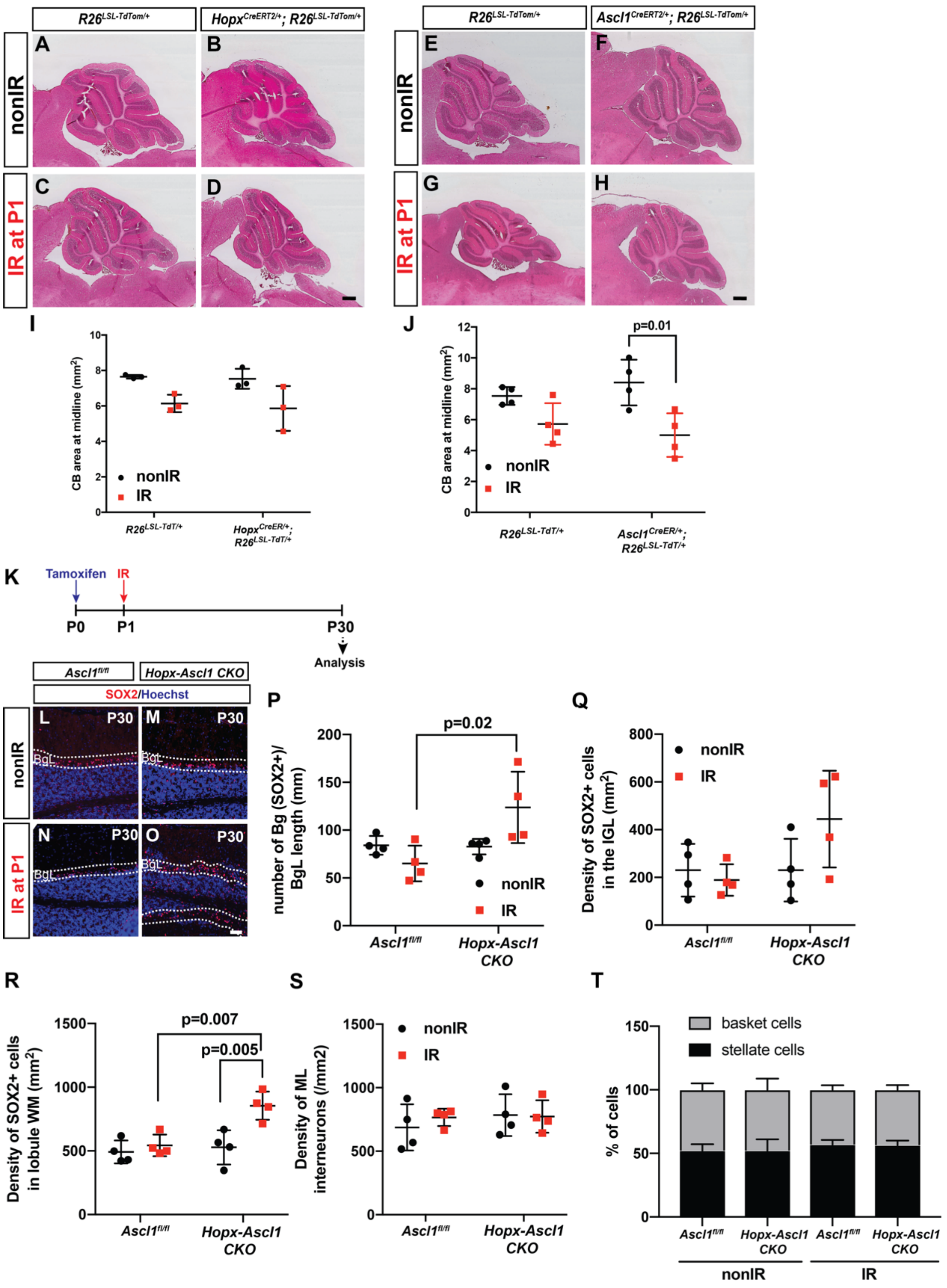

Fig. S13. **Loss of *Ascl1* in *Hopx*-NEPs increases Bg and astrocyte production upon irradiation but does not alter ML interneuron density. A-J.** H&E analysis and the quantification of the cerebellar area at the midline of nonIR and IR, Cre+ and Cre-

littermates of *Hopx-TdT* (A-D, I, n=3/condition, Two-way ANOVA, $F_{(1,4)}$=13.41, p=0.02) and *Ascl1-TdT* (E-H, J, n=4/condition, Two-way ANOVA, $F_{(1,12)}$=17.26, p=0.001) animals at P30. **K.** Experimental plan. **L-O.** IF analysis of control and *Hopx-Ascl1* CKO nonIR and IR animals at P30 for SOX2 (Bg and astrocytes). **P-R.** Quantification of the density of SOX2+ Bg (P, n=4/condition, Two-way ANOVA, $F_{(1,12)}$=6.878, p=0.02), and density of astrocytes in the IGL (Q, n=4/condition, Two-way ANOVA, p=0.086), and lobule WM (R, n=4/condition, Two-way ANOVA, $F_{(1,12)}$=10.56, p=0.007). **S-T.** Quantification of ML interneuron density (S, n=4/condition, Two-way ANOVA, p=0.4) and the distribution of Basket vs. Stellate neurons (T, n=4/condition) in nonIR and IR *Hopx-Ascl1* CKO animals and control littermates shows no differences. Scale bars: A-H: 500µm, L-O: 100µm, BgL: Bergmann glia layer

List of Tables and Additional Supplementary Materials

Movie 1. Live imaging of the lobule 3 of a P7 nonIR *Ascl1-TdT* cerebellum that was given Tm at P5. Outline shows external granule layer. Time lapse images are shown in Fig. S8.

Movie 2. Live imaging of the lobule 3 of a P7 IR *Ascl1-TdT* cerebellum that was IR at P1 and given Tm at P5. Outline shows external granule layer. Time lapse images are shown in Fig. S8.

Table S1. Binomial test results showing the cell-type specific genes of the clustering analysis shown in Figs. 1, 3, S1.

Table S2. Differential expression analysis amongst N1 and N6 (related to Fig. S2).

Table S3. Summary of the distribution of the TdT+ cells after long term GIFM in *Ascl1-TdT* brains that were given Tm at P0 or P5 (related to Fig. 2).

Table S4. Summary of the distribution of the TdT+ cells after long term GIFM in *Hopx-TdT* brains that were given Tm at P0 or P5 (related to Fig. 2).

Table S5. Summary of the results of GIFM in *Ascl1-TdT* and *Hopx-TdT* brains in the deep white matter (related to Figs. S6, S9, S12)

Table S6. GSEA results of the differentially expressed genes between the nonIR and IR N1 *Hopx*+ NEPs and N6 *Ascl1*+ NEPs and NEPs in clusters N2-5 (related to Fig. S7)

Table S7. Differential expression analysis between nonIR and IR cells in cluster N1 *Hopx*+ NEPs and N6 *Ascl1*+ NEPs (related to Fig. 3, Fig. S5)

Table S8. Top genes that are co-expressed with *Ascl1* in nonIR and IR cells (related to Fig. 3)

Table S9. Summary of the distribution of the TdT+ cells after long term genetic inducible fate mapping in nonIR and IR *Ascl1-TdT* brains that were given Tm at P0 or at P5 (related to Fig. 5)

Table S10. List of antibodies.

Table S11. Sequences complementary to the probes used for RNA *in situ* hybridization.

Table S12. Summary of the statistics.

Tables

Table S3. Summary of the distribution of the TdT+ cells after long term genetic inducible fate mapping in *Ascl1-TdT* brains that were given Tm at P0 or at P5 (related to Fig. 2)

	**% cells in TdT+ cells at P30**	
**Cell type**	**Tm at P0 (n=4)**	**Tm at P5 (n=3)**
Granule cells	0.66 ± 0.48	0 ± 0
ML Interneurons	95.10 ± 2.34	97.01 ± 4.45
Bergmann glia	0.26 ± 0.13	0 ± 0
IGL Astrocyte	1.37 ± 0.83	0.92 ± 1.61
WM Astrocyte	2.15 ± 1.06	0.99 ± 1.55
WM oligodendrocyte	0.46 ± 0.15	1.06 ± 1.31

Table S4. Summary of the distribution of the TdT+ cells after long term genetic inducible fate mapping in *Hopx-TdT* brains that were given Tm at P0 or at P5 (related to Fig. 2)

	**% cells in TdT+ cells at P30**	
**Cell type**	**Tm at P0 (n=3)**	**Tm at P5 (n=3)**
Granule cells	2.03 ± 1.33	1.06 ± 0.07
ML Interneurons	31.23 ±1.31	1.09 ± 0.29
Bergmann glia	51.02 ± 3.26	70.37 ± 2.71
IGL Astrocyte	10.53 ±1.20	20.3 ± 3.10
WM Astrocyte	4.11 ± 1.02	5.27 ± 1.08
WM oligodendrocyte	0.84 ±0.42	1.26 ± 0.99
Purkinje cells	0.23 ± 0.40	0.65 ± 0.26

Table S5. Summary of the results of GIFM in *Ascl1-TdT* and *Hopx-TdT* brains in the deep white matter.

**GIFM results in the deep WM**		**Tm at P0**		**Tm at P5**	
		**2 days after Tm**	**P30**	**2 days after Tm**	**P30**
Hopx-TdT	**nonIR**	no labelling	no labelling	no labelling	no labelling
	**IR at P1**	no labelling	no labelling	no labelling	no labelling
Ascl1-TdT	**nonIR**	SOX2+ cells	Mostly astrocytes and rare oligodendrocytes	no labelling	no labelling
	**IR at P1**	SOX2+ cells	Mostly astrocytes and rare oligodendrocytes	no labelling	Mostly astrocytes and rare oligodendrocytes

Table S9. Summary of the distribution of the TdT+ cells after long term genetic inducible fate mapping in nonIR and IR *Ascl1-TdT* brains that were given Tm at P0 or at P5 (related to Fig. 5)

	**% cells in TdT+ cells at P30**			
	**Tm at P0 (n=3)**		**Tm at P5 (n=3)**	
**Cell type**	**nonIR (n=4)**	**IR at P1 (n=3)**	**nonIR (n=5)**	**IR at P1 (n=4)**
ML Interneurons	95.41 ± 2.36	88.58 ± 0.79	96.28 ± 4.75	81.23 ± 4.57
Granule cells	0.69 ±0.48	0.69 ± 0.41	1.0 ± 1.4	11.83 ± 2.00
Bergmann glia	0.26 ± 0.13	0.04 ± 0.03	0 ± 0	0.35 ± 0.29
IGL Astrocyte	1.42 ± 0.83	5.75 ± 0.76	0.95 ± 1.60	1.06 ± 0.61
WM Astrocyte	2.21 ± 1.06	4.94 ± 1.51	1.04 ± 1.55	5.52 ± 1.86

Table S10. List of antibodies

**Target**	**Catalog Number**	**Company**	**Dilution**
goat α-SOX2	AF2018	R&D System	1/200 (adult)-<br>1/500 (pups)
rabbit α-PAX2	71600	Invitrogen	1/500
rabbit α-PAX6	AB2237	Millipore	1/500
rat α-GFP (CFP)	04404-84	Nacalai Tesque	1/1000
mouse α-ASCL1	556604	BD Pharmingen	1/1000
guinea pig α-PVALB	195-004	Synaptic Systems	1/1000
rabbit α-S100B	Z0311	DAKO	1/1000
mouse α-NeuN	MAB3777	Millipore	1/1000
rabbit α-HOPX	HPA030180	Sigma (Prestige Ab)	1/1000

Table S11. Sequences complementary to the probes used for RNA *in situ* hybridization.

Gene	Sequence (5’-3’)
Hopx	TCTAGATAACCCTCACTAAAGATCTCCGGAGGCAGCACTTGAGGCGCTTCCTCAGTATACTGTCCCCTCGGAGTGTCAG<br>GCGGGAGGCGTCTTCTTCCTCCTCTCCATCCTTAGTCAGACGCGCACGGACCATGTCGGCGCAGACCGCGAGCGGCCC<br>CACGGAGGACCAGGTGGAGATCCTGGAGTACAACTTCAACAAGGTCAACAAGCACCCGGACCCCACCACGCTGTGCCT<br>CATCGCAGCCGAGGCGGGTCTCACGGAGGAGCAGACGCAGAAATGGTTTAAGCAGCGCCTGGCAGAGTGGCGGCGGT<br>CAGAAGGCTTGCCTTCGGAATGCAGATCTGTTACGGACTAGGGAGCCAGGCCCTTGAGCTTGCTCTTGGAACTCCATCT<br>CTTCTTCCTTCCCTCGGCTTACCCTATAGTGTCACCTAAATCCCGGG
Gdf10	GTTCCAGACAAGATGAACTCCCTTGGAGTCCTTTTCCTGGATGAAAATCGGAATGCGGTTCTGAAGGTGTACCCCAATAT<br>GTCCGTAGAGACCTGTGCCTGTCGGTAAGATGGCTTCAAGATAGAAGACAGACCTGCTTCATCCCTGCCCTGCAGAGTG<br>GCAATCTTGGAGCCAGGGCTTGACTCGGGGAGGTTCCAGGTGCTAGACAGAGCTTACAGGCAGCCCTGCTGGGACCAA<br>GAAAGATCTGCCCACCACATCGCAATTCTTCAGTTCTTCCGTGCTGGTGGTAGCTCTGTAAAGACGTGTTGAGTTCCTGG<br>AAGAAATCTGGAATTAACTGTGGTCTGCAATTTGCCCATCATCCCTGCCCACACTTTTCAAGGCCTAGAAATAACGTGTGT<br>CCTCAAATGTCAAC
Igfbp5	ACCACCACCCCCAATTCTGACCCGATCCCGCCTGGGGGTTTCTACGGTCTCCGCTCGCTCTGCGTGCACCTGGCGCGC<br>CTCTTTTTTTCACCCCCAACCTGTTGCAAGTCTTTAATCCTCGCAATTGGGACTTGCGTGCAGGCATCTGAATCCTCCTTG<br>CCTCATATTTTGCAAGTGTTTGGGGGAGAGCACCTGCTCTACCTGCAAGAGATTTAAAAGGAAAAAAATCTCCAGGCTCC<br>CTCTTTCTCCACACACTCTCGCTCTCCTGCCCCGCCCCGAGGTAAAGCCAGACTCCGAGAAAATGGTGATCAGCGTGGT<br>CCTCCTGCTGCTGGCCGCCTATGCCGTACCGGCTCAAGGCCTGGGTTCTTTCGTGCACTGTGAACCCTGCGACGAGAAA<br>GCTCTGTCCATGTGTCCCCCCAGCCCTCTGGGCTGTGAGCTGGTCAAAGAGCCCGGCTGTGGCTGCTGCATGACTTGC<br>GCCCTGGCGGAGGGACAGTCGTGTGGCGTCTACACGGAGCGCTGCGCCCAGGGTTTGCGCTGCCTCCCCCGGCAGGA<br>TGAGGAGAAGCCGCTGCACGCCCTGCTGCACGGCCGCGGGGTTTGCCTCAACGAAAAGAGCTACGGCGAGCAAACCAA<br>GATAGAGAGAGACTCTCGGGAACACGAGGAACCCACCACCTCCGAGATGGCTGAAGAGACCTACTCCCCCAAGGTCTTC<br>CGGCCCAAGCACACTCGCATTTCCGAGCTGAAGGCTGAGGCTGTGAAGAAGGACCGCAGAAAGAAGCTGACCCAGTCC<br>AAGTTTGTGGGGGGTGCAGAGAACACTGCCCACCCCAGAGTCATCCCTGCACCTGAGATGAGACAGGAATCCGAACAA<br>GGC
Grin3a	CATCGTCAGCTCAGAAAACAACTTTTTCATCTGGAACTTGCAGTATGACCCTATGGGAAAGCCAATGTGGACTCGCCTGG<br>GTAGCTGGCAAGGGGGAAGGATTGTCATGGACTCGGGAATATGGCCAGAGCAGGCCCAGAGGCACAAAACCCACTTCC<br>ATCACCCAAACAAGTTACACTTGAGAGTGGTGACACTGATTGAACATCCATTTGTTTTCACGAGAGAAGTAGATGATGAAG<br>GCTTATGCCCTGCTGGACAACTCTGTCTAGACCCTATGACTAATGACTCTTCCATACTGGATAGCCTGTTTAGCAGCCTAC<br>ATAGCAGTAATGATACAGTGCCAATCAAGTTCAAGAAGTGCTGCTATGGGTATTGCATTGATCTACTGGAACAGTTAGCA<br>GAAGACATGAACTTTGACTTTGACCTCTATATTGTAGGGGATGGAAAGTATGGAGCTTGGAAAAATGGTCACTGGACTGG<br>GCTGGTTGGTGATCTCCTGAGTGGAACAGCCAACATGGCAGTCACTTCTTTCAGCATCAATACTGCAAGAAGCCAAGTGA<br>TAGATTTCACCAGCCCTTTCTTCTCAACCAGTTTGGGCATCTTAGTGAGGA
Arntl	CAACCTCAGCTGCCTCGTTGCAATCGGGCGCCTGCACTCGCACATGGTTCCACAACCAGCGAACGGGGAAATACGGGT<br>GAAATCTATGGAGTACGTTTCTCGACACGCAATAGATGGGAAATTTGTTTTTGTAGATCAGAGGGCGACAGCTATTTTGG<br>CGTATCTACCACAGGAACTTCTAGGTACATCATGTTATGAGTATTTTCATCAAGACGACATAGGACACCTCGCAGAATGTC<br>ACAGGCAAGTTTTACAGACAAGAGAAAAGATCACGACTAATTGCTATAAGTTTAAGATCAAAGATGGTTCTTTTATCACGC<br>TACGAAGTCGATGGTTCAGTTTCATGAACCCGTGGACCAAGGAAGTTGAATACATTGTCTCAACCAACACTGTTGTTTTAG<br>CCAATGTCCTGGAAGGCGGGGACCCAACCTTCCCGCAGCTAACAGCACCCCCCCACAGCATGGACAGCATGCTGCCCT<br>CTGGAGAAGGTGGCCCAAAGAGGACTCATCCCACTGTCCCAGGCATTCCAGGGGGAACCAGAGCCGGAGCAGGAAAAA<br>TAGGTCGAATGATCGCCGAGGAAATCATGGAAATCCACAGGATAAGAGGGTCATCGCCTTCCAGCTGTGGCTCCAGCCC<br>GCTGAACATCACAAGTACGCCTCCCCCTGATGCCTCTTCTCCAGGAGGCAAGAAGATTCTAAATGGAGGGACTCCAGAC<br>ATTCCTTCCACTGGACTATTACCAGGGCAGGCTCAGGAGACCCCAGGGTA

Table S12. Summary of the statistics performed.

Figure	Test Performed	p-value	Multiple comparisons	
Figure 3B	Two-way ANOVA	$F_{(1,15)}$=25.75, p=0.0001	P5	p=0.001
Figure 4D	Two-way ANOVA	All layers: $F_{(7,42)}$=8.718, p<0.0001	Granule neurons IGL nonIR vs. IGL IR	p=0.004
Figure 4G	Two-way ANOVA	All layers: $F_{(1,14)}$=5.047, p=0.04	Granule neurons IGL nonIR vs. IGL IR	p=0.04
Figure S9J	Two-tailed Student's t-test	EGL: t=5.5, df=4 p=0.005	N/A	
Figure S9K	Two-way ANOVA	$F_{(1,20)}$=19.5, p=0.0003	ML+BgL	p=0.002
Figure S9K	Two-tailed Student's t-test	EGL: t=7.05 df=5 p=0.0009		
Figure S10F	Two-way ANOVA	All layers: $F_{(3,32)}$=12.89, p<0.0001	EGL 48h nonIR vs. IR	p=0.008
			BgL 48h nonIR vs. IR	p=0.007
Figure S10F	Two-way ANOVA	EGL only: $F_{(1,8)}$=5.94, p=0.04	24h IR vs. 48h IR	p=0.03
			48h nonIR vs. 48h IR	p=0.003
Figure S10H	Two-way ANOVA	treatment: $F_{(3,20)}$=31.28, p<0.0001	EGL	p=0.0001
			ML+BgL	p=0.03
Figure S12B	Two-way ANOVA	By layer and condition $F_{(7,14)}$= 137.9, p<0.0001	ML+BgL (BgL-like)	p=0.03
			Granule neurons IGL nonIR vs. IGL IR	p=0.0001
			Astrocyte WM nonIR vs. WM IR	p=0.2
Figure S12B	Student's t-test for WM astrocytes	t=4.966, df=4, p=0.007	N/A	
Figure S12D	Two-way ANOVA	$F_{(7,83)}$=275.4 p<0.0001	Post hoc multiple comparisons are not significant.	

Figure 6M	Two-tailed Student's t-test	t=5.83, df=4 p=0.004	N/A	
Figure 6N	Two-way ANOVA	$F_{(1,8)}$=7.928, p=0.02	SOX2 IR vs. ASCL1 IR	p=0.02
			ASCL1 nonIR vs. ASCL1 IR	p=0.002
Figure 7J	Two-way ANOVA	by treatment: $F_{(1,19)}$=78.43, p<0.0001 by genotype: $F_{(1,19)}$=14.39, p<0.001	control nonIR vs. IR	p=0.0003
			mutant nonIR vs. IR	p<0.0001
			control IR vs. mutant IR	p=0.004
Figure 7S	Two-way ANOVA	by treatment: $F_{(1,10)}$=13.11, p=0.005 by genotype: $F_{(1,10)}$=16.41, p=0.002	Control nonIR vs. IR	p=0.004
			Control IR vs. mutant IR	p=0.001
Figure 7T	Two-way ANOVA	by treatment: $F_{(1,15)}$=9.134, p=0.009 by genotype: $F_{(1,15)}$=58.49, p<0.0001	control nonIR vs. IR	p=0.00001
			mutant nonIR vs. IR	p=0.03
Figure 7T	Two-way ANOVA	by treatment: $F_{(1,15)}$=9.134, p=0.009 by genotype: $F_{(1,15)}$=58.49, p<0.0001	control IR vs. mutant IR	p=0.002
			Control nonIR vs. IR	p=0.00001
			Mutant nonIR vs. IR	p=0.03
Figure 7U	Two-way ANOVA	by genotype: $F_{(1,15)}$=6.549, p=0.02	control IR vs. mutant IR	p=0.001
Figure S13I	Two-way ANOVA	treatment: $F_{(1,12)}$=17.26, p=0.0013	Post hoc multiple comparisons are not significant.	

Figure S13J	Two-way ANOVA	treatment: $F_{(1,12)}=17.26$, $p=0.0013$	Mutant nonIR vs. IR	p=0.01
Figure S13P	Two-way ANOVA	by genotype: $F_{(1,12)}=6.878$, $p=0.02$	control IR vs. mutant IR	p=0.02
Figure S13Q	Two-way ANOVA	by genotype: $F_{(1,12)}=3.488$, $p=0.086$	N/A	
Figure S13R	Two-way ANOVA	by condition: $F_{(1,12)}=12.49$ $p=0.004$ by genotype: $F_{(1,12)}=10.56$ $p=0.007$	mutant nonIR vs. IR	p=0.005
			control IR vs. mutant IR	p=0.007
Figure S13S	Two-way ANOVA	by genotype: $F_{(1,12)}=0.534$ $p=0.4$	N/A	
